# Overall, emotional and social loneliness and their associated factors: a large-scale cross-sectional study of young and middle-aged adults in Germany

**DOI:** 10.1186/s12889-026-30122-4

**Published:** 2026-10-29

**Authors:** Pauline Kleinschlömer, Helena Ludwig-Walz, Sabine Diabaté, Martin Bujard

**Affiliations:** 1 Federal Institute for Population Research (BiB), Wiesbaden, Germany; 2 Institute of Medical Psychology, University Heidelberg, Heidelberg, Germany

**Keywords:** Loneliness, Emotional loneliness, Social loneliness, Young adults, Middle-aged adults, Migration background, Mental health, Social relationships, Personality traits, Public health.

## Abstract

**Background:**

Loneliness is increasingly recognized as a major public health concern, yet little is known about the distinct patterns and associated factors of emotional and social loneliness during young and middle adulthood. This study examined age-related differences in overall, emotional, and social loneliness and associated sociodemographic, social, health-related, and personality factors among adults in Germany.

**Methods:**

We analysed nationally representative data from the German Family Demography Panel Study (FReDA), including 14,443 adults aged 18 to 51 years. Loneliness was assessed using the validated De Jong Gierveld Loneliness Short Scale, distinguishing overall, emotional, and social loneliness. Multivariable regression models examined associations with sociodemographic, social, health-related, and personality factors. Analyses were stratified by age and gender.

**Results:**

High levels of loneliness were common, with 24.3% of participants reporting intense emotional loneliness, 29.6% intense overall loneliness, and 35.8% intense social loneliness. Overall loneliness remained relatively stable across age groups. In contrast, emotional loneliness was lower in older age groups, while social loneliness was higher. Social relationships showed some of the strongest associations with loneliness, particularly social loneliness: discussing personal matters within one’s social network (β = -0.32, 95% CI -0.37 to -0.28) and having a partner (β = -0.21, 95% CI -0.26 to -0.16) were associated with lower social loneliness, while first-generation migration background was associated with higher social loneliness (β = 0.31, 95% CI 0.24 to 0.38). Mental health burdens were consistently associated with higher loneliness, especially emotional loneliness, with depressed mood showing the strongest association (β = 0.22, 95% CI 0.17 to 0.27). Among personality traits, agreeableness was associated with lower social loneliness (β = -0.19, 95% CI -0.22 to -0.17), whereas neuroticism was associated with higher emotional loneliness (β = 0.17, 95% CI 0.14 to 0.20). Gender differences were generally small, as were associations with physical health conditions.

**Conclusions:**

Loneliness in young and middle adulthood is multidimensional, with emotional and social loneliness showing distinct age-related patterns and associated factors. Social relationships showed some of the strongest associations with lower loneliness, while mental health burdens and first-generation migration background were associated with higher loneliness. Public health strategies may benefit from considering different loneliness dimensions and psychosocial contexts.

**Supplementary Information:**

The online version contains supplementary material available at https://doi.org/10.1186/s12889-026-30122-4.

## Background

Loneliness - defined as the subjective perception of insufficient quality or quantity of social relationships [1, 2] - is increasingly recognised as a public health concern [3, 4]. It is associated with adverse outcomes including poorer physical and mental health and premature mortality [5–10].

Most empirical studies have treated loneliness as a unidimensional construct, typically focusing on overall loneliness as the primary outcome [5, 11–13]. Within this unidimensional literature, findings on age-related patterns are inconsistent, ranging from U-shaped and other non-linear trajectories to linear declines or stable levels [13–19], potentially reflecting differences in measurement, study design, and national or cultural context [12, 13, 20–22]. Compared with research on older adults, young and middle adulthood has received less attention in loneliness research, although this life phase (often described as the “rush hour of life” [23]) is marked by career development, family formation, and changes in social roles and relationships, which may shape experiences of loneliness [22, 24]. A recent German panel underscores the relevance of loneliness in this population, with approximately one-third of young and middle-aged adults reporting overall loneliness [25].

However, the predominantly unidimensional approach to loneliness may provide only a partial picture. A multidimensional approach may therefore offer a more comprehensive understanding of loneliness by distinguishing between emotional and social loneliness [26–28]. This distinction builds on Weiss’s relational perspective, which states that emotional loneliness reflects unmet needs for intimacy, attachment, and emotional support, while social loneliness reflects unmet needs for broader social integration, companionship, and network support [27]. Importantly, these dimensions may differ in their correlates and consequences [28].

From a theoretical perspective, the distinction between emotional and social loneliness can be further informed by Granovetter’s theory of the “strength of weak ties” [29]. Granovetter distinguishes between strong ties, such as close friends, partners, or family members, which are characterised by emotional intimacy and frequent contact, and weak ties, such as acquaintances, colleagues, or more peripheral social contacts, which connect individuals to broader social networks and social structures. Applied to loneliness research, deficits in strong ties may be particularly relevant to emotional loneliness, whereas deficits in weak ties may contribute to social loneliness through reduced integration into wider social networks. This perspective provides a theoretical rationale for expecting different associations between relational characteristics and emotional and social loneliness. At the same time, Cacioppo’s conceptualisation of loneliness [2, 30] emphasises that loneliness depends on individuals’ subjective evaluations of their relationship quality rather than on objective network characteristics alone. Together, these perspectives suggest that both the structure and perceived quality of social relationships may contribute differently to emotional and social loneliness.

Previous research has linked loneliness to a broad range of factors, including sociodemographic characteristics, social relationships, physical and mental health, and personality traits [7, 8]. However, most evidence concerns overall loneliness, and considerably less is known about whether these factors are differentially associated with emotional and social loneliness.

Sociodemographic characteristics may be relevant to loneliness because they reflect differences in social, economic, and life-course circumstances that may shape opportunities for social participation, relationship formation and access to social resources. However, evidence for dimension-specific associations remains heterogeneous. Education, for example, has shown inconsistent associations, ranging from no association with either dimension [31], to lower social loneliness among highly educated younger adults [32], and modest associations in older adults [33]. Recent evidence from young and middle-aged adults in Germany further suggests that socioeconomic resources may be relevant to both dimensions, with income poverty associated with higher and income wealth with lower emotional and social loneliness [34]. Migration-related experiences may be relevant to loneliness, as migration can disrupt established social networks and involve barriers to social integration [35–37]. These processes may affect both emotional and social loneliness, although existing evidence regarding dimension-specific associations remains inconsistent [32, 35]. Importantly, migration background encompasses heterogeneous experiences, with recent longitudinal evidence from Germany suggesting variation by gender and age at migration [38].

Social relationship factors may be particularly relevant to distinguishing emotional and social loneliness because different relationships fulfil different relational needs. Consistent with the distinction between strong and weak ties [29], close relationships may be particularly relevant to emotional loneliness, whereas broader social connections may be more relevant to social loneliness. Empirically, partnership has particularly been associated with lower emotional loneliness [31–33], whereas having children has not been associated with either dimension [32]. Recent evidence links overall loneliness to perceived support from friends, companionship within personal networks, and perceived social support. Longitudinal studies further indicate that the relevance of different social network ties may change throughout adulthood [39–41]. However, research into how broader social networks specifically relate to emotional and social loneliness remains limited.

Health-related factors may also be associated with loneliness because mental and physical health can shape individuals’ experiences of social relationships and opportunities for social participation. In a representative German population sample, overall loneliness was associated with a broad range of mental health indicators, including depressive and anxiety symptoms, perceived stress, and lower life satisfaction [16]. Recent evidence further implies that the relationship between loneliness and mental health may be bidirectional [10, 42]. Dimension-specific evidence suggests that poor mental health is associated with both emotional and social loneliness, although associations may be stronger for emotional loneliness [32, 43]. Physical health may be particularly relevant when functional limitations restrict social participation. Recent German research has shown associations with both dimensions, with particularly pronounced differences in social loneliness among middle-aged adults [44]. Together, these findings suggest that both mental and physical health should be considered when examining the correlates of different loneliness dimensions.

Personality traits may likewise be relevant as they are related to how individuals establish, maintain, and perceive social relationships. Recent evidence supports robust associations between loneliness and the Big Five personality traits (extraversion, agreeableness, conscientiousness, neuroticism, and openness), particularly higher levels of neuroticism and lower levels of extraversion [45–47]. Further, longitudinal studies have found that there are links between personality traits and subsequent loneliness [47]. However, evidence distinguishing emotional from social loneliness remains limited. Among Norwegian students, neuroticism was positively associated with both emotional and social loneliness, whereas other traits were negatively associated [48]. It remains unclear whether these dimension-specific associations also apply across young and middle adulthood.

Building on the conceptual distinction between emotional and social loneliness and the evidence for potentially dimension-specific correlates outlined above, we registered two interrelated research questions and six corresponding hypotheses [49]. Using large-scale, nationally representative data from German adults aged 18 to 51 years, we first examine the prevalence of overall, emotional, and social loneliness in young and middle adulthood, hypothesising that the three forms of loneliness vary by age (H1) and gender (H2). Second, we hypothesise that sociodemographic characteristics (H3), social relationships (H4), health-related factors (H5), and personality traits (H6) are differentially associated with overall, emotional and social loneliness. In addition, we examine whether these associations vary by age and gender.

## Methods

### Dataset and sample

We use data from the German Family Demography Panel Study (FReDA) [50, 51], a large-scale representative survey of the German population aged 18–51. For our study, we focus on wave 1B (autumn/winter 2021/22), which includes 20,216 respondents. Wave 1B includes most of our variables of interest. However, information on educational background is included in wave 1R (the recruitment wave). Depressed mood and partnership status are available in wave 1 A (spring/summer 2021), and Big Five personality traits are included in wave 2B (autumn/winter 2022/23). We have therefore transferred these variables to wave 1B. This is straightforward for variables obtained from waves 1R and 1 A, as all FReDA subwaves of wave 1 contribute jointly to wave 1 [52]. The Big Five personality traits, however, were measured after the loneliness outcomes in wave 2B. Their inclusion is supported by evidence indicating that personality traits remain largely stable over periods of up to four years [53, 54], which suggests that the later measurement approximates respondents’ dispositions at the time of the loneliness assessment. This does not establish temporal ordering. We therefore interpret all associations as cross-sectional and do not make claims about the direction of effects. We excluded respondents who identified their gender as diverse (*N* = 46) due to the insufficient sample size, which did not allow for separate analyses. Analyses were conducted on complete cases, resulting in the exclusion of respondents with missing values for any of the variables of interest (*N* = 5,727). This results in a final sample size of *N* = 14,443. Around two-thirds of the missing information was concentrated in the Big Five personality variables, which consist of 15 items and are therefore more prone to partial nonresponse. To assess the robustness of our findings, we additionally performed multiple imputation for the Big Five variables (see Supplement Tables A4-A6). Because the main analyses rely on complete cases, we also compared included and excluded participants. Excluded participants reported higher levels of social and emotional loneliness, were more likely to have a low level of education and to be first-generation migrants. Further, they reported lower satisfaction with their household income. The standardised differences were small to modest (|Cohen’s d| ≤ 0.23). The differences on most other characteristics, including the Big Five traits, depressed mood and anxiety disorders, were negligible (see Supplement Table A20).

### Variables

Loneliness: Overall loneliness, emotional loneliness and social loneliness are our dependent variables. The measurement is based on the De Jong Gierveld Loneliness Short Scale (DJGLS), which is validated for several age groups and countries (26). Emotional loneliness is measured with the questionnaire items “(1) I experience a general sense of emptiness”; “(2) I miss having people around”; “(3) I often feel rejected”. The items “(4) There are plenty of people I can rely on when I have problems”; “(5) There are many people I can trust completely” and “(6) There are enough people I feel close to” are used to measure social loneliness (see FReDA questionnaire and scale overview [50]. The answer categories range from one to three, covering (“yes”, “more or less” and “no”). Processing the scale we follow the validated operationalisation by De Jong Gierveld [26, 55]. This entails counting the neutral (“more or less”) and positive (“yes”) answers on items 1, 2 and 3 for the emotional loneliness score. The neutral (“more or less”) and negative (“no”) answers on items 4, 5, and 6 are counted for the social loneliness score. Hence, for both dimensions the score ranges from zero (“not lonely”) to three (“intensely lonely”). The internal consistency, assessed using McDonald’s Omega, was acceptable for both dimensions (social loneliness: ω = 0.85; emotional loneliness: ω = 0.71). The overall loneliness score was calculated by summing the emotional and social loneliness scores, following the approach by De Jong Gierveld [26, 55], resulting in a total score ranging from 0 to 6. To facilitate graphical comparison of trends in emotional and social loneliness (each ranging from 0 to 3), with the trend in overall loneliness (ranging from 0 to 6), we divide the overall loneliness score by two. This rescaling results in a range from 0 ("not lonely") to 3 ("intensely lonely") across all three loneliness dimensions studied in this paper. The distribution of respondents across each value is robust for both the original overall loneliness scale (ranging from 0 to 6) and the adjusted overall loneliness scale (ranging from 0 to 3) (see Supplement Table A1). To enable comparability across the three dimensions of loneliness, we conducted an analysis using standardised (z‑score) values. The results remain robust (see Supplement Tables A12 - A14).

Associated Factors: To examine factors associated with loneliness, we analysed four distinct sets of variables, which capture socio-demographic characteristics, social relationships, health-related variables, and personality traits (Big Five). Table 1 provides an overview of these sets, their corresponding dimensions, and operationalisation. The selection of variables was guided by prior research on factors associated with overall loneliness and the existing research gaps regarding emotional and social loneliness. Our aim was to test whether these factors, well documented for overall loneliness, apply differently to emotional and social loneliness. We also checked for multicollinearity among the variables; correlations ranged from -0.35 to 0.28 (see Supplement Table A2). The mean variance inflation factor across all predictors is 1.92, suggesting minimal risk of bias due to shared variance among the predictors.

We started with the *base model* that includes age (in categories 18–25, 26–30, 31–35, 36–40, 41–45, 46–51) and binary gender, as both have consistently been discussed in the literature as relevant correlates of loneliness [13, 15, 17–19, 56]. We categorized age into groups rather than modelling it continuously, as this allows for a flexible representation of the age-loneliness relationship without imposing a linear functional form, given the mixed evidence on the shape of age-related loneliness patterns in prior research.

The first extended set covered *socio-demographic variables*, with higher socio-economic status consistently associated with lower overall loneliness [13, 17, 34, 57]. Household financial security (“Making ends meet”) was assessed using respondents’ subjective evaluation on a six-point scale, ranging from [1] “great difficulties” to [6] “very good”. Educational background was classified according to the International Standard Classification of Education (ISCED) 2011 and summarised on a three-point scale: low (ISCED 0–2), middle (ISCED 3–4), and high (ISCED 5–8). Migration was also considered, given mixed evidence on its role in loneliness across different age groups and loneliness dimensions [31–33, 35]. We distinguish between respondents with no migration background and those who are first- or second-generation migrants (see Table 1). Respondents of the first generation have immigrated to Germany themselves. Respondents of the second generation were born in Germany, but at least one parent immigrated to Germany.

The second set captured *social relationship variables*, repeatedly associated with lower overall loneliness [13, 16, 17, 21, 57]. These include partnership status (single, living-apart-together, cohabiting), as evidence highlights this variable as one of the factors most strongly associated with lower overall loneliness [13, 16, 17, 21, 57], while the association has been mainly described with emotional loneliness [31–33]. The role of having children remains unclear across all dimensions of loneliness [32] in the current literature and was incorporated as a yes/no variable. Furthermore, the role of respondents’ social networks (measured by whether personal matters are discussed with friends, acquaintances, neighbors, or colleagues; yes/no) is insufficiently researched. Current literature supports an association between friend-based social support and lower loneliness [39–41]; however, its association with emotional and social loneliness remains unclear. Following Granovetter’s distinction between strong and weak ties, partnership status and broader social network indicators were conceptually mapped onto different types of social relationships.

The third set included *health-related variables*, as both mental and physical conditions have consistently been associated with overall loneliness [7, 13, 16] and partly with emotional and social loneliness [32]. Mental health was captured through anxiety disorder (established medical diagnoses, self-reported) and depressed mood, assessed by asking about past-week experiences ([1] “never” to [4] “most/all of the time.”), which was recoded into a binary variable ([1] and [2] = [0] “no”; [3] and [4] = [1] “yes”). Overall life satisfaction was measured with the validated 11-point Likert scale [58], given that higher life satisfaction has been associated with lower loneliness [16]. However, evidence regarding its association with emotional and social loneliness is scarce [59]. Physical health was assessed based on common diseases in the target population according to the Global Burden of Disease Study [60, 61], including type 2 diabetes, hypertension, cancer, and asthma (all self-reported medical diagnoses). These indicators were included to examine associations between physical health and loneliness in young and middle adulthood, given heterogeneous and partly inconclusive evidence in prior studies [7, 13, 32], particularly with respect to diagnosis-based indicators and emotional and social loneliness dimensions. Obesity was classified using the World Health Organization’s (WHO) Body Mass Index (BMI) threshold of ≥ 30 [62]. In addition, two variables addressed perceived burden and positive experiences during the COVID-19 pandemic, as data collection occurred during the later phase of the pandemic and potential effects could not be excluded [63–67].

The fourth variable set comprised the Big Five *personality traits*, each of which has been associated with loneliness in previous research [45, 48]. We applied the validated Big Five Inventory – 2 Extra Short Form (BFI-2-XS) [68]. Instead of the 60-item BFI-2 scale, the BFI-2 Extra Short Form assesses each of the five personality domains (extraversion, agreeableness, conscientiousness, neuroticism and openness to experience) with three items - one per facet - resulting in a total of 15 items. Although its brevity is known to reduce internal consistency compared to the full scale (McDonald’s Omega ranging from ω 0.43 to ω 0.68 across domains), the BFI-2-XS has been validated for domain-level assessment, and our large sample size provides sufficient statistical power to detect meaningful associations [69]. Response options for all items ranged from [1] “not at all” to [5] “absolutely” (see Table 1). The full item battery can be found in the supplement (Supplement Table A3).

**Table 1:** Overview of variable sets, dimensions and variables

Variable Sets	Dimension	Operationalisation	
		Variable	Values
Base Model	Age	Age categories (wave 1B)	18–25
			26–30
			31–35
			36–40
			41–45
			46–51
	Gender	Gender (binary) (wave 1B)	(1) men
			(2) female
Socio-Demographic Variables	Income	Subjective evaluation of household financial security (Making ends meet) (wave 1B)	(1) great difficulties
			(6) very good
	Educational background	ISCED Categories (wave 1R)	(1) low: ISCED 0–2
			(2) middle: ISCED 3–4
			(3) high: ISCED 5–8
	Migration Background	Migration Background (wave 1B)	(0) no migration background
			(1) 1st generation migrant
			(2) 2nd generation migrant
Social Relationships	Children	Respondent has children (wave 1B)	(0) no
			(1) yes
	Partnership	Partnership Status (wave 1 A)	(1) single
			(2) Living Apart Together
			(3) cohabitation
	Social Network	Discuss personal matters with friends, acquaintances, neighbours, and colleagues (wave 1B)	(0) no
			(1) yes
Health-Related Variables	Mental Health	Doctor has diagnosed anxiety disorder (wave 1B)	(0) no
			(1) yes
		Depressed Mood (wave 1 A)	(0) no
			(1) yes
		Life Satisfaction (wave 1B)	(0) not at all satisfied
			(10) completely satisfied
	Physical Health	Doctor has diagnosed type 2 diabetes (wave 1B)	(0) no
			(1) yes
		Doctor has diagnosed hypertension (wave 1B)	(0) no
			(1) yes
		Doctor has diagnosed cancer (wave 1B)	(0) no
			(1) yes
		Doctor has diagnosed asthma (wave 1B)	(0) no
			(1) yes
		Obesity (self-reported BMI) (wave 1B)	(0) BMI < 30 (1) BMI > = 30
	COVID-19 Pandemic	Personally heavily burdened (wave 1B)	(1) strongly disagrees
			(5) strongly agree
		Can also see a positive aspect (wave 1B)	(1) strongly disagrees
			(5) strongly agree
Personality	Big Five Inventory	Extraversion (wave 2B)	(1) not at all
			(5) absolutely
		Agreeableness (wave 2B)	(1) not at all
			(5) absolutely
		Conscientiousness (wave 2B)	(1) not at all
			(5) absolutely
		Neuroticism (wave 2B)	(1) not at all
			(5) absolutely
		Openness to new experiences (wave 2B)	(1) not at all
			(5) absolutely

*Source*: FReDA Panel

### Analyses

The study’s research questions, hypotheses (H1- H6), and analysis plan were registered on the Open Science Framework (OSF) [49]. Analyses not specified in the registration were considered exploratory.

We employed multiple ordinary least squares (OLS) regressions to examine the occurrence of three dimensions of loneliness – overall, emotional, and social – across age groups and gender, as well as their associations with different sets of variables. As a robustness check, given the ordinal and polarised nature of the loneliness measures, we re-estimated our main models using ordered logit and computed the expected value of the outcome scale. The resulting predictions were virtually identical to those from the OLS models (Supplement Table A21). Separate regression models were estimated for each loneliness dimension. For each dimension, the variable sets were included stepwise in the following order: (1) age and gender (base model) (2), socio-demographic variables (3), social relationship variables (4), health-related variables, and (5) personality traits. Variables were entered stepwise to assess the contribution of the different variable sets. This stepwise approach allowed us to assess the unique contribution of each variable set while accounting for potential overlaps and interdependencies.

To illustrate predicted levels of loneliness by age and gender, we calculated marginal means, estimating values for each age category and sex while holding all other variables at their sample means. These estimates were used to produce the graphical trends shown in the results.

### Statistics and reproducibility

The analyses were conducted using STATA 18. The code used for the analyses will be made publicly available on the OSF upon publication.

## Results

### Characteristics of participants

The final sample comprised 14,443 participants (mean age of 34.8 years) with a near-equal gender distribution (49.8% male, 50.2% female). 29.6% of respondents report intense overall loneliness, representing the highest score on the 0–3 loneliness scale. The prevalence of intense loneliness is higher for social loneliness (35.8%). While both overall and social loneliness are fairly evenly distributed across genders, females report intense emotional loneliness slightly more often than males, with a prevalence in the full sample of 24.3% (see Table 2).

**Table 2:** Prevalence and Gender Composition of Individuals Experiencing Intense Loneliness (overall, emotional and social loneliness)

Full Sample	Intense overall loneliness	Intense emotional loneliness	Intense social loneliness
	29.57%	24.28%	35.80%
Gender			
Female	14.13%	13.3%	17.72%
Male	15.44%	10.98%	18.08%

Percentages under gender indicate the share of individuals reporting intense loneliness in each category (overall, social, emotional). Intense loneliness corresponds to a score of 3 on a scale ranging from 0 to 3

*Source*: FReDA Panel, wave 1B (autumn/winter 2021/22). Weighted prevalence. Authors’ own calculations

These prevalence rates reflect extreme loneliness scores, while mean values capture the full distribution (see Table 3). Respondents’ ratings of overall loneliness (mean = 1.52) and emotional loneliness (mean = 1.49) are fairly evenly distributed across the 0 to 3 scale. Social loneliness, however, is more polarised (mean = 1.56), with many scoring 0 (31.0%) or 3 (35.8%). The frequency and mean values for variables relating to sociodemographic characteristics, social relationships, health and personality traits are listed in Table 3.

**Table 3:** Overview of Participant Characteristics

	Variables			
		Proportions/ Mean (SD)	Min	Max
Loneliness Variables	Overall Loneliness	Mean: 1.52	0	3
	0 (Not lonely)	11.9%		
	0.5	17.0%		
	1	14.3%		
	1.5	14.6%		
	2	12.7%		
	2.5	12.8%		
	3 (Intensely Lonely)	16.8%		
	Emotional Loneliness	Mean: 1.49	0	3
	0 (Not Lonely)	22.8%		
	1	29.8%		
	2	23.1%		
	3 (Intensely Lonely)	24.3%		
	Social Loneliness	Mean:1.56	0	3
	0 (Not lonely)	31.0%		
	1	17.8%		
	2	15.3%		
	3 (Intensely Lonely)	35.9%		
Base Model	Age	Mean: 34.77	18	51
	18–25	20.1%		
	26–30	14.9%		
	31–35	17.1%		
	36–40	16.8%		
	41–45	15.5%		
	46–51	15.5%		
	Sex		0	1
	Male	49.8%		
	Female	50.2%		
Socio-Demographic Variables	Subjective Evaluation of HH Financial Security	4.43 (1.26)	1	6
	Educational Background		1	3
	Low	10.7%		
	Middle	56.3%		
	High	33.0%		
	Migration Background		0	2
	No Migration Background	72.1%		
	1. Generation Migrant	16.2%		
	2. Generation Migrant	11.7%		
Social Relationships	Children		0	1
	No Children	52.6%		
	Kids	47.4%		
	Partnership Status		1	3
	Single	28.9%		
	Living Apart Together	12.2%		
	Cohabitation	58.9%		
	Discuss Personal Matters with Social Network		0	1
	No	39.3%		
	Yes	60.7%		
Health-Related Variables	Anxiety		0	1
	No	89.3%		
	Yes	10.7%		
	Depressed Mood		0	1
	No	85.7%		
	Yes	14.3%		
	Life Satisfaction	7.21 (1.87)	0	10
	Type 2 Diabetes		0	1
	No	97.4%		
	Yes	2.6%		
	Hypertension		0	1
	No	89.1%		
	Yes	10.9%		
	Cancer		0	1
	No	98.2%		
	Yes	1.8%		
	Asthma		0	1
	No	91.1%		
	Yes	8.9%		
	Obesity		0	1
	No	82.2%		
	Yes	17.8%		
	COVID-19 Pandemic: See a Positive Side	3.50 (1.13)	1	5
	COVID-19 Pandemic: Negatively Affected	3.20 (1.17)	1	5
Big Five	Extraversion	3.03 (0.74)	1	5
	Agreeableness	3.63 (0.73)	1	5
	Conscientiousness	3.65 (0.78)	1	5
	Neuroticism	2.95 (0.65)	1	5
	Openness to Experience	3.30 (0.79)	1	5

*N* = 14,443. *Source*: FReDA Panel, wave 1B (autumn/winter 2021/22); educational background from wave 1R, depressed mood and partnership status from wave 1A, Big Five personality traits from wave 2B (autumn/winter 2022/23). Descriptive statistics are weighted. Authors’ own calculations

### Base model estimates of overall, emotional, and social loneliness

**Fig. 1:**
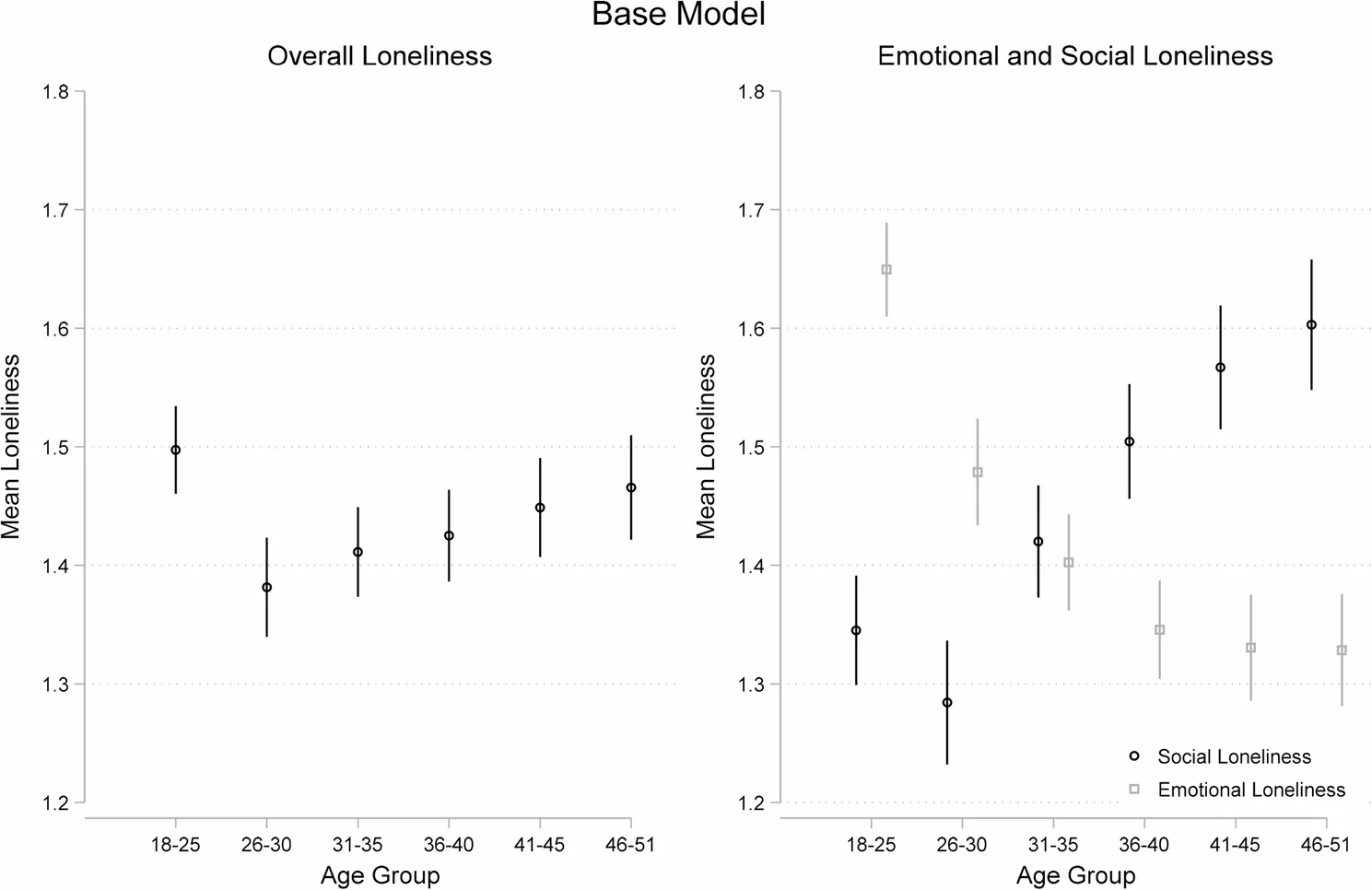
Base Model for Overall Loneliness, Emotional Loneliness and Social Loneliness (*n* = 14,443). *Note*: Base model includes age (categorical) and gender (binary). Loneliness was measured on a scale ranging from 0 to 3. To enhance interpretability, the y-axis was restricted to the observed value range; the full scale is provided in Supplement Figure A1 with corresponding regression table in Supplement Table A7. *Source*: FReDA Panel, wave 1B (autumn/winter 2021/22). Authors’ own calculations

Figure 1 presents mean levels of overall, emotional, and social loneliness across age groups from the base model, which includes age and gender only. Overall loneliness was most pronounced among 18-25-year-olds (*mean: 1.50; 95% CI: 1.46*, *1.53*, *p* < .001), lower in the 26–30 age group (*mean: 1.38; 95% CI: 1.34*, *1.42*, *p* < .001), and somewhat higher again in older age groups, with a mean of *1.47* (*95% CI: 1.42*, *1.51*, *p* < .001) in the 46-51-year-olds. Emotional loneliness was highest in the youngest group (18–25 years; *mean: 1.65; 95% CI: 1.61*, *1.69*, *p* < .001) and lower in the 26–30 group, with successively lower values in subsequent age groups, reaching the lowest levels in the 46–51 years age group (*mean: 1.33; 95% CI: 1.28*, *1.38*, *p* < .001). Levels were relatively stable thereafter. In contrast, social loneliness was lowest among 18-25-year-olds (*mean: 1.35; 95% CI: 1.30*, *1.39*, *p* < .001) and 26-30-year-olds (*mean: 1.28; 95% CI: 1.23*, *1.34*, *p* < .001), and higher in older groups, with the highest mean observed in the 46–51 age group (*mean: 1.60; 95% CI: 1.55*, *1.66*, *p* < .001; see Supplement Table A7).

### Factors associated with overall, emotional, and social loneliness

In the next step, the variable sets (1) socio-demographic variables (2), social relationships (3), health-related variables, and (4) personality traits were added sequentially to the base model. The stepwise regression results for all included variables are provided in Supplement Tables A8 – A10. Table 4 represents the regression coefficients and 95% CI for all variables included in the fully adjusted model, grouped by covariate domain.

Social relationships showed the most consistent negative associations with loneliness across all dimensions, with the strongest associations observed for social loneliness (e.g., social network: *β: -0.32; 95% CI: -0.37*, *-0.28*, *p* < .001, * R² = 0.15*; partnership [living apart together]: *β: -0.23; 95% CI: -0.29*, *-0.16*, *p* < .001, *R² = 0.15*; partnership [cohabitation]: *β: -0.21; 95% CI: -0.26*,* -0.16*, *p* < .001, *R² = 0.15*). Life satisfaction showed a stable, though less pronounced, negative association with all loneliness dimensions (*β: -0.12; 95% CI: -0.13*, *-0.11*, *p* < .001, *R² = 0.20 for overall loneliness*, *R² = 0.19 for emotional loneliness*, *R² = 0.15 for social loneliness*). Older age (from approximately 36 years onward) was associated with lower levels of emotional loneliness (*β ranging from − 0.14 to -0.12*, *p* < .001). Among the Big Five personality traits, agreeableness showed the most pronounced association with lower levels of social (*β: -0.19; 95% CI: -0.22*, *-0.17*, *p* < .001, * R² = 0.15*) and overall loneliness (*β: -0.12; 95% CI: -0.14*,* -0.10*, *p* < .001, *R² = 0.20*). A better subjective evaluation of household financial security was associated with lower levels of loneliness (*β ranging from − 0.07 to -0.06*, *p* < .001). Additionally, perceiving positive aspects during the COVID-19 pandemic was negatively associated with loneliness, although effect sizes were small (*β ranging from − 0.04 to -0.02*, *p* < .01).

A first-generation migration background was positively associated with higher levels of social (*β: 0.31; 95% CI: 0.24*, *0.38*, *p* < .001, *R² = 0.15*) and overall loneliness (*β: 0.21; 95% CI: 0.15*, *0.26*, *p* < .001, *R² = 0.20*). In contrast, second-generation migrants showed substantially smaller associations, as exemplified by social loneliness (*β: 0.08; 95% CI: 0.01*, *0.14*, *p* = .014, *R² = 0.15*). Indicators of mental health burden (depressed mood and anxiety disorders) were consistently associated with higher levels of loneliness across all dimensions, particularly in emotional loneliness [(depressed mood: *β: 0.22; 95% CI: 0.17*, *0.27*, *p* < .001, *R² = 0.19*); (anxiety disorder: *β: 0.11; 95% CI: 0.06*, *0.17*, *p* < .001, *R² = 0.19*)]. Age showed a positive association with social loneliness, with stronger associations observed in older age groups (*β ranging from 0.18 to 0.32*, *p* < .001). Among the Big Five, neuroticism was positively associated with all loneliness dimensions, with the comparatively highest association with emotional loneliness (*β: 0.17; 95% CI: 0.14*, *0.20*, *p* < .001, * R² = 0.19*). In addition, personal burden experienced during the COVID-19 pandemic was positively associated with emotional loneliness (*β: 0.13; 95% CI: 0.11*, *0.14*, *p* < .001, *R² = 0.19*) and, to a lesser extent, overall loneliness (*β: 0.07; 95% CI: 0.06*, *0.08*, *p* < .001, *R² = 0.20*). Finally, female gender was positively associated with emotional (*β: 0.09; 95% CI: 0.05*, *0.12*, *p* < .001, *R² = 0.19*) and overall loneliness (*β: 0.04; 95% CI: 0.01*, *0.07*, *p* < .01, *R² = 0.20*).

Associations of educational attainment, parental status, and most physical health indicators with any dimension of loneliness were small, with estimates generally close to zero and CIs including the null value (Table 4, Supplement Tables A8 – A10). With regard to explained variance (*R²*), the health status variable set contributed notably to the variance in overall and emotional loneliness. Further details are presented in Supplement Tables A8 – A10.

Overall, the most consistent and comparatively pronounced associations across all three loneliness dimensions were observed for partnership status, social network, depressed mood, and life satisfaction, although their magnitude varied across the dimensions. Several associations were more dimension-specific: Social network, partnership status and first-generation migration background were particularly strongly associated with social loneliness, whereas depressed mood, partnership status and life satisfaction were more strongly associated with emotional loneliness. Across the fully adjusted models, the comparatively large associations were in the range of approximately 0.2 to 0.3 points on the loneliness scale ranging from 0 to 3, while many other statistically detectable associations were below 0.1 scale points and were therefore small in substantive terms.

**Table 4:** Factors Associated with Overall, Emotional, and Social Loneliness (*n* = 14,443)

Variable Sets	Predictor		Overall loneliness β [95% CI]	Emotional loneliness β [95% CI]	Social loneliness β [95% CI]
Base Model	Age	18-25	Ref.	Ref.	Ref.
		26-30	0.00 [-0.05; 0.05]	-0.03 [-0.09; 0.03]	0.04 [-0.03; 0.10]
		31-35	0.06 [0.01; 0.12]*	-0.06 [-0.12; 0.00]	0.18 [0.11; 0.25]**
		36-40	0.06 [0.00; 0.12]*	-0.12 [-0.19; -0.06]**	0.24 [0.17; 0.32]**
		41-45	0.08 [0.02; 0.14]*	-0.14 [-0.21; -0.08]**	0.29 [0.21; 0.37]**
		46-51	0.09 [0.03; 0.15]*	-0.14 [-0.21; -0.07]**	0.32 [0.24; 0.40]**
	Gender	men	Ref.	Ref.	Ref.
		female	0.04 [0.01; 0.07]*	0.09 [0.05; 0.12]**	-0.00 [-0.04; 0.04]
Socio-Demographic Variables	Income	1-6, higher = greater satisfaction	-0.06 [-0.08; -0.05]**	-0.06 [-0.07; -0.04]**	-0.07 [-0.09; -0.05]**
	Educational background	low: ISCED 0-2	Ref.	Ref.	Ref.
		middle: ISCED 3-4	0.00 [-0.09; 0.09]	0.05 [-0.05; 0.15]	-0.04 [-0.16; 0.07]
		high: ISCED 5-8	-0.08 [-0.17; 0.01]	-0.03 [-0.13; 0.07]	-0.12 [-0.24; -0.01]*
	Migration Background	no migration background	Ref.	Ref.	Ref.
		1^st^ generation migrant	0.21 [0.15; 0.26]**	0.10 [0.05; 0.16]**	0.31 [0.24; 0.38]**
		2^nd^ generation migrant	0.05 [0.00; 0.09]*	0.02 [-0.03; 0.07]*	0.08 [0.01; 0.14]*
Social Relationships	Children	no	Ref.	Ref.	Ref.
		yes	0.01 [-0.03; 0.05]	-0.01 [-0.05; 0.04]	0.02 [-0.03; 0.07]
	Partnership	single	Ref.	Ref.	Ref.
		Living Apart Together	-0.18 [-0.23; -0.13]**	-0.14 [-0.19; -0.08]**	-0.23 [-0.29; -0.16]**
		cohabitation	-0.18 [-0.23; -0.14]**	-0.16 [-0.21; -0.12]**	-0.21 [-0.26; -0.15]**
	Social Network	no	Ref.	Ref.	Ref.
		yes	-0.20 [-0.23; -0.16]**	-0.07 [-0.10; -0.03]**	-0.32 [-0.37; -0.28]**
Health-Related Variables	Mental Health	Anxiety: no	Ref.	Ref.	Ref.
		Anxiety: yes	0.11 [0.06; 0.16]**	0.11 [0.06; 0.17]**	0.10 [0.04; 0.17]*
		Depressed mood: no	Ref.	Ref.	Ref.
		Depressed mood: yes	0.19 [0.14; 0.24]**	0.22 [0.17; 0.27]**	0.16 [0.10; 0.22]**
		0-10, higher = greater satisfaction	-0.12 [-0.13; -0.11]**	-0.12 [-0.13; -0.11]**	-0.12 [-0.13; -0.11]**
	Physical Health	Diabetes type 2: no	Ref.	Ref.	Ref.
		Diabetes type 2: yes	-0.05 [-0.15; 0.04]	-0.06 [-0.16; 0.05]	-0.05 [-0.18; 0.07]
		Hypertension: no	Ref.	Ref.	Ref.
		Hypertension: yes	0.02 [-0.04; 0.07]	-0.01 [-0.07; 0.05]	0.04 [-0.02; 0.11]
		Cancer: no	Ref.	Ref.	Ref.
		Cancer: yes	0.03 [-0.08; 0.14]	0.04 [-0.08; 0.16]	0.02 [-0.12; 0.16]
		Asthma: no	Ref.	Ref.	Ref.
		Asthma: yes	0.05 [-0.00; 0.10]	0.04 [-0.01; 0.10]	0.06 [-0.01; 0.12]
		Obesity (BMI<30): no	Ref.	Ref.	Ref.
		Obesity (BMI≥30): yes	0.00 [-0.04; 0.05]	-0.02 [-0.06; 0.03]	0.02 [-0.03; 0.08]
	COVID-19 Pandemic	Positive Aspects: 1-5, higher = greater disagreement	-0.03 [-0.04; -0.02]**	-0.02 [-0.04; -0.01]*	-0.04 [-0.05; -0.02]*
		Personal Burden: 1-5, higher = greater disagreement	0.07 [0.06; 0.08]**	0.13 [0.11; 0.14]**	0.01 [-0.01; 0.03]
Personality	Big Five Inventory	1-5, higher = greater neuroticism	0.12 [0.10; 0.15]**	0.17 [0.14; 0.20]**	0.07 [0.04; 0.10]**
		1-5, higher = greater openness	-0.02 [-0.04; 0.00]	0.01 [-0.01; 0.03]	-0.04 [-0.07; -0.02]*
		1-5, higher = greater agreeableness	-0.12 [-0.14; -0.10]**	-0.05 [-0.07; -0.02]**	-0.19 [-0.22; -0.17]**
		1-5, higher = greater conscientiousness	-0.05 [-0.07; -0.03]**	-0.07 [-0.09; -0.04]**	-0.04 [-0.07; -0.02]**
		1-5, higher = greater extraversion	-0.05 [-0.07; -0.03]**	-0.02 [-0.05; 0.00]	-0.08 [-0.11; -0.05]**
Socio-demographic variables	Social relationships	Health status	Personality (Big Five)		

Coefficients and 95% CI (confidence intervals) are based on Model 4, which includes socio-demographic variables, social relationships, health status, and personality traits

*Source:* FReDA Panel, wave 1B (autumn/winter 2021/22); educational background from wave 1R, depressed mood and partnership status from wave 1A, Big Five personality traits from wave 2B (autumn/winter 2022/23). Authors’ own calculations

* *p*<0.05, ** *p*<0.001

### Full model estimates of overall, emotional, and social loneliness

**Fig. 2:**
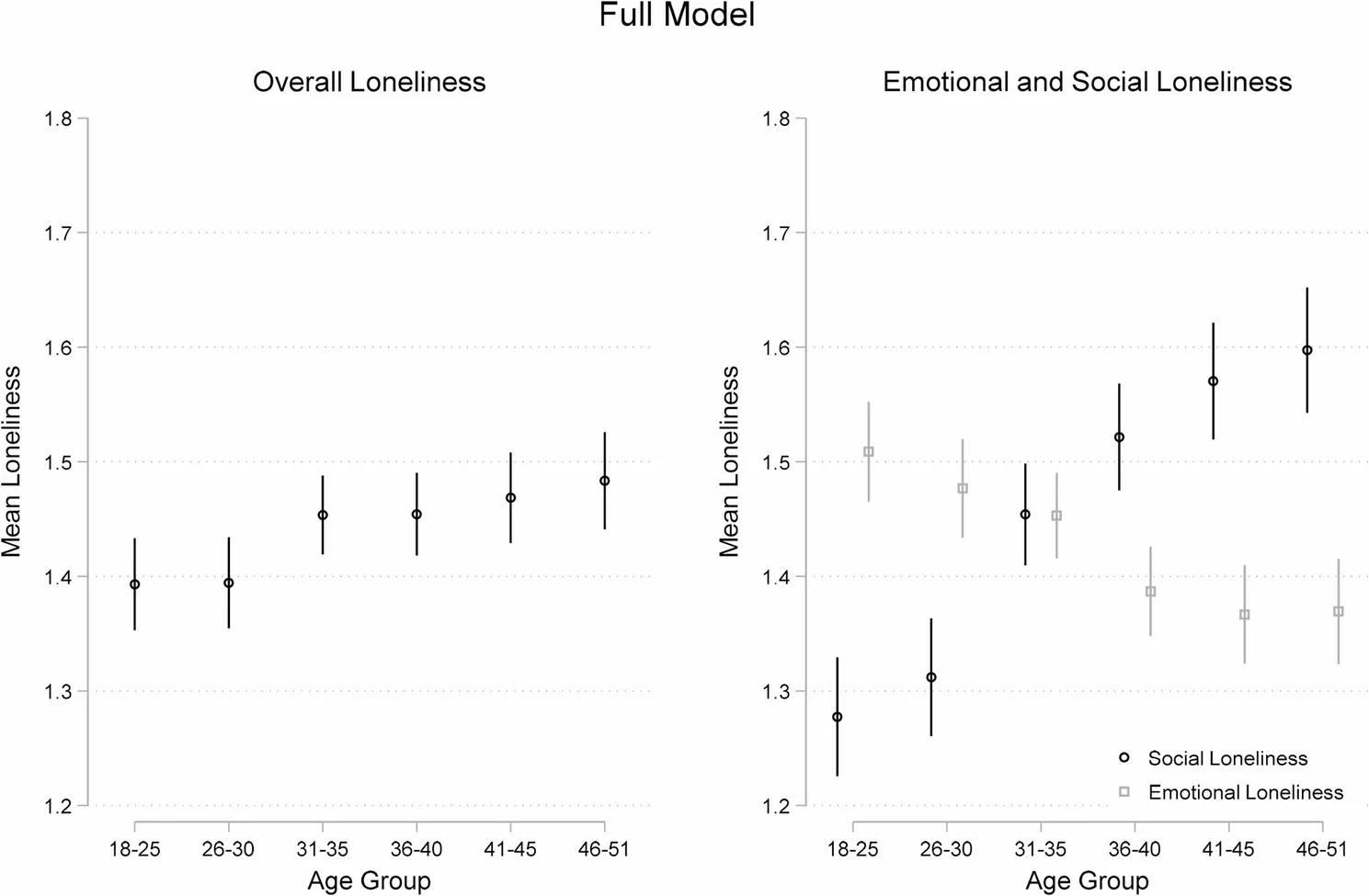
Full Model for Overall Loneliness, Emotional Loneliness and Social Loneliness (*n* = 14,443). *Note*: Full model including age (categorical) and gender (binary), and all variable sets from Models 1-4: socio-demographic characteristics, social relationships, health variables, and personality traits. Loneliness was measured on a scale ranging from 0 to 3. To enhance interpretability, the y-axis was restricted to the observed value range; the full scale is provided in Supplement Figure A2 with corresponding regression table in Supplement Table A11. *Source*: FReDA Panel, wave 1B (autumn/winter 2021/22); educational background from wave 1R, depressed mood and partnership status from wave 1A, Big Five personality traits from wave 2B (autumn/winter 2022/23). Authors’ own calculations

Next, we stratified each loneliness dimension again by age in the fully adjusted model to analyse whether the inclusion of the variables was associated with differences in loneliness across age groups. The results show that the previously observed higher mean of overall loneliness among individuals aged 18–25 years in the base model was less pronounced in the fully adjusted model, resulting in only minor age-related differences (*ranging from 1.39 to 1.48*, *p* < .001; Fig. 2, Supplement Table A11). However, differences across age groups in emotional and social loneliness remained, though at a reduced magnitude. Emotional loneliness was highest among 18-25-year-olds (*mean: 1.51; 95% CI: 1.47–1.55*, *p* < .001) and showed lower means in older age groups, with the lowest mean observed in the 46-51-year age group (*mean: 1.37; 95% CI: 1.32–1.42*, *p* < .001). Conversely, social loneliness was lowest in the youngest age group (*mean: 1.28; 95% CI: 1.23–1.33*, *p* < .001) and was higher in older groups, with the highest mean observed in the 46-51-year group (*mean: 1.60; 95% CI: 1.54–1.65*, *p* < .001).

### Loneliness by gender: overall, emotional, and social loneliness

**Fig. 3:**
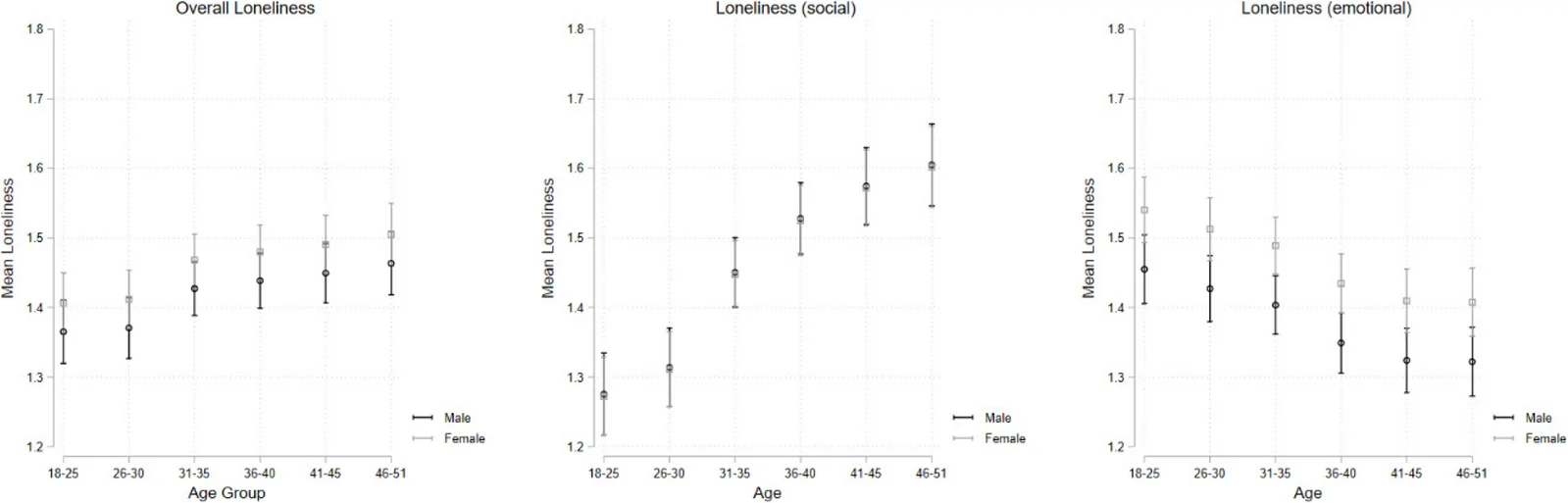
Comparing Loneliness by Gender: Overall, Emotional, and Social Loneliness (female *n* = 8,009; male *n* = 6,427). *Note*: Full model including age (categorical) and gender (binary), and all variable sets from Models 1-4: socio-demographic characteristics, social relationships, health variables, and personality traits. Loneliness was measured on a scale ranging from 0 to 3. To enhance interpretability, the y-axis was restricted to the observed value range; the full scale is provided in Supplement Figure A3, with corresponding regression tables in Supplement Tables A13-A14. *Source*: FReDA Panel, wave 1B (autumn/winter 2021/22); educational background from wave 1R, depressed mood and partnership status from wave 1A, Big Five personality traits from wave 2B (autumn/winter 2022/23). Authors’ own calculations

Gender-stratified associations were generally small, and estimates were largely similar between women and men (Fig. 3, Supplement Table A15). For emotional loneliness there is a tendency for women to report higher levels than men, with significant differences in the 31–35 and 36–40 age groups. In gender-specific models of associated predictors, associations were generally similar between men and women, with the clearest gender difference observed for ‘openness to experience’. Openness showed a small negative association with social loneliness in men (*β: -0.09; 95% CI: -0.12*,* -0.05*, *p* < .001), whereas the estimate was close to zero in women (*β: -0.01; 95% CI: -0.05*, *0.03*, *p* = .64). Overall, gender-specific differences in predictor associations were limited, with openness to experience representing the clearest isolated difference. Detailed results are available in Supplement Tables A16-17.

### Loneliness by age: overall, emotional, and social loneliness

For each loneliness dimension, we additionally tested age-group differences in the three most relevant predictors identified in the full model: depressed mood, partnership status, and life satisfaction for emotional loneliness, and migration background, partnership status, and social network for social loneliness. Across these predictors, heterogeneity by age group emerged only for partnership status: For emotional loneliness, being in a partnership (LAT and cohabitation) was associated with significantly lower loneliness only among respondents in middle adulthood (age groups 41–45 and 46–51), with no significant difference between partnered and single individuals in the younger age groups. For social loneliness, by contrast, partnered respondents showed significantly lower loneliness not only in the oldest age group [46–51] but also among 26-30- and 31-35-year-olds. Overall, age-related heterogeneity in predictor associations was limited and emerged primarily for partnership status, whereas the remaining associations were broadly consistent across age groups. Detailed results are available in Supplement Tables A18-A19.

## Discussion

This study provides evidence on the age- and gender-related distribution of overall, emotional and social loneliness and their associated variables among adults aged 18 to 51 in Germany. Three key findings emerge. First, intense loneliness was common with prevalence rates ranging from 24.3% for emotional loneliness to 29.6% for overall loneliness and 35.8% for social loneliness. Second, emotional and social loneliness showed opposing age-related patterns: emotional loneliness was lower in older age groups, whereas social loneliness was higher. In contrast, overall loneliness varied little across age groups. Third, social relationships and mental health-related factors showed the most consistent associations across loneliness dimensions, although the magnitude of these associations varied. Social networks and first-generation migration background showed particularly pronounced associations with social loneliness, whereas depressed mood was most strongly associated with emotional loneliness. These findings underscore the conceptual distinction between loneliness dimensions and the importance of considering their correlates. Given the large sample size, we emphasise the magnitude and precision of estimated associations, as reflected by regression coefficients (β) and their 95% CIs, rather than statistical significance alone. Small coefficients are interpreted cautiously even when their CIs exclude the null value [70, 71].

Approximately one-quarter to one-third of young and middle-aged adults in our study reported high levels of emotional, social or overall loneliness, assessed using the validated DJGLS [26, 55]. Although comparisons with previous research are limited by differences in measures and study designs, longitudinal evidence from Germany indicates that loneliness increased during the COVID-19 pandemic, particularly among adolescents and young adults. Although levels have since declined, they appear to remain above pre-pandemic levels [72]. As our data were collected during the COVID-19 pandemic, the observed prevalence may partly reflect these exceptional circumstances. Nevertheless, previous research suggests that associations between loneliness and sociodemographic, social and psychological factors are also observed beyond the pandemic context [7].

Regarding age-related differences, overall loneliness varied little across the age groups examined, consistent with several large-scale studies reporting relatively small differences in loneliness among middle-aged adults [13, 14, 21, 73]. However, other studies have reported higher loneliness in young adulthood than in older age groups [13, 14, 73]. In our analyses, age-related differences in overall loneliness substantially attenuated after adjustment for social relationship variables (Model 2). The relatively small differences observed across age groups are broadly aligned with the meta-analysis by Mund et al. [21], which synthesised 75 longitudinal studies across the adult life course. Importantly, these authors identified the lack of differentiation between emotional and social loneliness as a key limitation of the existing literature. By distinguishing between these dimensions, our study revealed opposing age-related patterns that were not apparent when examining overall loneliness alone. Emotional loneliness was lower in older age groups, whereas social loneliness was higher. Early adulthood is often characterised by transitions such as leaving home, starting work or education, and forming new social ties, alongside processes of identity development and increasing autonomy. These circumstances may coincide with differences in social connectedness, perceived exclusion and unmet relational needs [19, 74], although our cross-sectional data do not allow us to examine such processes directly. Previous studies differentiating loneliness dimensions during young and middle adulthood are limited. Existing evidence generally indicates lower emotional loneliness in older age groups [31, 32, 75, 76], consistent with our findings. In contrast, evidence on social loneliness is more heterogeneous, with studies reporting higher levels in older groups [32, 76], while others suggest relative stability [75] or lower levels [31]. These findings illustrate how analyses of overall loneliness alone may obscure distinct age-related patterns in emotional and social loneliness.

Gender differences in overall loneliness were small, with women reporting slightly higher levels than men. This aligns with prior research indicating that gender differences, when observed, tend to be small and inconsistent in direction [13, 16, 77–79], potentially varying by measurement instrument used (e.g. single-item vs. multi-item scales) [11]. A meta-analysis by Maes et al. [78], including 751 effect sizes from nearly 400,000 individuals, reported negligible gender differences (Hedges’ g = 0.07). In our dimension-specific analyses, emotional loneliness was slightly higher among women aged 31–40; however, the magnitude of this difference was small. Previous findings on gender differences in emotional and social loneliness are similarly mixed, with some studies reporting higher emotional loneliness among women [31, 33, 35], while others report higher social loneliness [32] and some identifying no consistent differences [48].

Social relationships emerged as particularly important correlates of loneliness. Social networks and partnership status were among the factors most consistently associated with lower loneliness across all three dimensions. This is in line with previous large-scale research highlighting the relevance of social and relational resources throughout adulthood [16, 17, 32, 40, 41]. However, the magnitude of these associations differed across dimensions. Social networks showed the strongest inverse association with social loneliness, a more modest negative association with overall loneliness and a relatively small association with emotional loneliness. Similarly, partnership status was more strongly associated with social loneliness than with overall or emotional loneliness. This contrasts with studies reporting stronger inverse associations between partnership and emotional loneliness [31–33, 35], indicating that associations between partnership and loneliness dimensions may differ across populations and contexts. Having children, by contrast, was not associated with lower loneliness, consistent with other studies reporting no systematic associations between parenthood and loneliness [17].

Migration background was another characteristic showing pronounced dimension-specific associations. Consistent with previous studies reporting higher loneliness among individuals with a migration background [35–37, 80], first-generation migration background was associated with higher social and overall loneliness, whereas its association with emotional loneliness was weaker. A Dutch study similarly identified a positive association between first-generation migration background and overall loneliness [35]. Recent longitudinal evidence from Germany further suggests heterogeneity in associations between migration background, loneliness and mental health, particularly by age at migration. For example, pronounced patterns have been reported among women who migrated to Germany after the age of 18 [38]. Possible contextual explanations discussed in the literature include the loss of close familial and cultural ties to one’s country of origin [81], experiences of exclusion, racism or discrimination [82] and barriers to establishing or maintaining broader social networks in the receiving country [38]. However, these mechanisms could not be examined in our study. Furthermore, our distinction between first- and second-generation migration background does not capture differences in age at migration, duration of residence, country of origin, language proficiency or cultural and social context. These characteristics should be considered when interpreting the observed associations. Notably, associations with second-generation migration background were substantially smaller, with estimates close to zero, especially for women.

Socioeconomic characteristics showed comparatively modest associations with loneliness. Previous studies linked higher educational attainment [13, 32, 33, 57] and higher income [13, 16, 34] to lower loneliness. In our study, however, associations between educational attainment and loneliness were generally small, with estimates close to zero across most dimensions. Household financial security showed a consistent but modest inverse association with all three loneliness dimensions.

Mental health-related factors were also consistently associated with loneliness, particularly its emotional dimension. Previous research has identified better physical and mental health [13, 16, 17, 32, 35, 57, 79] as well as higher life satisfaction and subjective well-being [12, 16, 57] as correlates of lower loneliness. However, relatively few studies have examined these associations separately for emotional and social loneliness while incorporating self-reported clinical diagnoses [32, 35]. In our analyses, depressed mood and anxiety disorders were meaningfully associated with higher loneliness across all dimensions, with the strongest associations observed for emotional loneliness, consistent with previous findings on psychological distress [32]. Life satisfaction was also consistently inversely associated with all loneliness dimensions. In contrast, individual physical health conditions, including hypertension, type 2 diabetes, asthma and obesity, showed generally small associations, with estimates close to zero and CIs frequently including the null value. This aligns with previous research reporting small or inconsistent associations between most physical health conditions and loneliness, particularly among younger populations [35]. For example, Hofman et al. (2022) reported weak associations of poor perceived health with both emotional and social loneliness and of higher body mass index with emotional loneliness, whereas associations with chronic diseases and functional limitations were small, inconsistent or non-significant [32]. Taken together, these findings suggest that physical health conditions may be less strongly associated with loneliness than psychosocial and mental health-related factors in young and middle adulthood. Stronger associations at later ages remain possible, particularly given the relatively low prevalence of severe physical illnesses in our comparatively young panel sample [60] and previous evidence on loneliness across the life course [9].

Further, our findings align with previous research on personality traits (Big Five), confirming that extraversion, agreeableness, conscientiousness, and (partially) openness are associated with lower levels of loneliness, while neuroticism remains consistently positively associated with all loneliness dimensions [45, 48]. Notably, in contrast to earlier studies, agreeableness showed the strongest negative association with loneliness in the fully adjusted models, particularly for social loneliness.

The observed differences between loneliness dimensions can be considered in light of Granovetter’s theory of strong and weak ties [29], which distinguishes emotionally close relationships associated with attachment needs from broader social connections associated with social integration and belonging. Consistent with this framework, social network ties showed the strongest association with social loneliness in our study. Migration background further supports a weak-tie interpretation: Having a first-generation migration background was more strongly associated with social than emotional loneliness. This pattern may be consistent with greater barriers to establishing or maintaining broader weak-tie networks in the host society, although the underlying mechanisms could not be examined in the present study. Individual characteristics also align with this pattern, as agreeableness was strongly associated with lower social loneliness but only weakly with emotional loneliness, suggesting that cooperative interpersonal styles may be conducive to maintaining broader weak-tie networks. In contrast, emotional loneliness was less clearly associated with strong-tie mechanisms. Partnership status was not more associated with emotional loneliness than with social loneliness, indicating that the presence of a partner alone may not be sufficient to offset emotional disconnection. Instead, emotional loneliness showed its strongest associations with psychological factors; depressed mood was particularly strongly associated with this dimension. Together, these findings suggest that emotional and social loneliness are associated with partially distinct relational and psychological factors, reinforcing the importance of a multidimensional, theory-informed approach.

As data collection took place during the COVID-19 pandemic (autumn/winter 2021/22), we additionally considered participants’ perceived pandemic-related experiences. Perceiving the pandemic as a personal burden was associated with higher emotional and overall loneliness, whereas reporting positive aspects was weakly associated with lower loneliness across all dimensions. These findings are broadly consistent with previous research linking pandemic-related stress to higher loneliness and resilience-related factors to lower loneliness [66]. However, the observed associations were considerably smaller than those of the main social and psychological correlates identified in our study. Thus, while pandemic-related experiences may be relevant to interpreting loneliness during the study period, social relationships and mental health-related factors remained the more pronounced correlates in our analyses.

Our findings have implications for future loneliness research and public health strategies. Loneliness is increasingly recognised as a public health concern by institutions such as the WHO [4], the UK government [83], and Germany’s national strategy [72, 84]. Nevertheless, evidence on the effectiveness of interventions remains limited. Psychological and social interventions have shown promising findings, particularly among older adults [73, 85–88], while evidence on digital approaches is mixed [87, 89, 90]. The methodological quality of existing intervention studies is often limited, and relatively few approaches explicitly distinguish between loneliness dimensions or are tailored to different age groups [73, 88]. In this context, the dimension-specific associations observed in our study suggest that distinguishing emotional and social loneliness may be relevant when developing and evaluating future interventions. Strengthening individuals’ “social convoy” through meaningful relationships, as discussed in theoretical approaches by De Jong Gierveld and Fokkema, has been proposed as a potential approach to supporting resilience [91].

Taken together, our findings underscore the multifactorial and context-sensitive nature of loneliness in young and mid-adulthood. Individuals without a partner or with limited social networks, those reporting mental health burdens, and people with a first-generation migration background may warrant particular attention in future research. The observed distinct age-related patterns of emotional and social loneliness also suggest that research may benefit from considering loneliness dimensions alongside age, social context, relationship status and psychological resources. This is particularly relevant given established associations between loneliness and adverse health outcomes, including depression, cardiovascular disease and all-cause mortality. Importantly, our observational findings do not establish whether interventions targeting the characteristics identified in this study would reduce loneliness. The effectiveness of dimension-specific or targeted approaches therefore requires investigation in appropriately designed intervention studies.

A major strength of our study is the multidimensional assessment of loneliness, distinguishing between emotional and social aspects, which is particularly relevant for young and middle adulthood. Drawing on large, representative panel data, we incorporated a broad set of validated measures (e.g., loneliness, Big Five personality traits, life satisfaction), along with detailed sociodemographic variables, health conditions, and migration background. However, some limitations need to be acknowledged. First, the cross-sectional design of our study limits causal interpretation and precludes differentiation between age and cohort effects, restricting conclusions about developmental trajectories over time [92]. Temporal ordering cannot be established, and bidirectional relationships are plausible, particularly for social relationships and mental health, as loneliness may itself be associated with social engagement, perceived relationship quality, and mental health. Additionally, the measures were not all collected concurrently. In particular, depressed mood and partnership status were assessed in Wave 1 A and the Big Five personality traits in Wave 2B, whereas the loneliness outcomes were assessed in Wave 1B. Although personality traits are relatively stable, they are not fixed, and loneliness may itself be related to later changes in self-reported personality. Depressed mood or partnership status may also fluctuate more strongly between subwaves. Nevertheless, identifying factors consistently associated with different loneliness dimensions can help characterise groups reporting higher or lower loneliness and inform hypotheses for future longitudinal and intervention research. In addition, the large, age-diverse sample (spanning nearly four decades) enables a comprehensive analysis of a wide range of relevant factors. Second, while many associations reached statistical significance, their magnitude varied substantially, and several estimates were small or close to zero. Explained variances (R²) were modest, which is common in population-based studies of psychosocial phenomena [93]. Third, additional limitations include the use of self-reported measures for income and BMI, which may not fully reflect objective experiences. In this vein, due to the ultra-short nature of the BFI-2-XS, internal consistency for the Big Five domains was modest. However, this is an expected trade-off between brevity and reliability [69]. Fourth, as data were collected during the COVID-19 pandemic, prevalence may reflect exceptional circumstances, which should be considered when interpreting generalisability. Additionally, as data collection for Wave 1 took place in spring/summer (subwave A) and autumn/winter (subwave B), and as these two subwaves were combined into one wave, different levels of pandemic severity at a macro level may have made the salience of the pandemic different across subwaves during data collection. Fifth, our analyses are based on complete cases and therefore exclude a significant proportion of the initial sample (*N* = 5,727 out of 20,216), primarily due to missing data on the Big Five items. Although multiple imputation analyses for the Big Five variables point to similar results, the analytic sample is not a random subset of the initial sample. Respondents who were excluded reported somewhat higher levels of loneliness, lower levels of education and lower satisfaction with their household income, and were more often first-generation migrants. While the differences were small to modest, they suggest that selection bias in the analytical sample cannot be ruled out. Consequently, mean levels of loneliness in our analytical sample may be slightly underestimated. Since the variance of the loneliness measures is almost identical in both groups, we consider it unlikely that the range will be substantially restricted, but we cannot exclude the possibility that some associations will differ in the excluded population. Lastly, as emotional and social loneliness items differ in wording direction (negative vs. positive), the results may be partly influenced by different response styles or mood activation, although we expect such bias to be small given the prior validation of the scale.

## Conclusion

In conclusion, this study advances the understanding of loneliness in young and middle adulthood by highlighting its multidimensional nature. Distinguishing between emotional and social loneliness revealed opposing age-related patterns that were not apparent when considering overall loneliness. Social relationships and mental health-related characteristics were consistently associated with loneliness, although the magnitude of associations differed across dimensions. Higher loneliness was particularly evident among individuals with limited social networks, those reporting mental health conditions, and those with a first-generation migration background. These findings highlight the relevance of considering loneliness dimensions alongside their psychosocial context in future research and the development and evaluation of public health strategies. Given the cross-sectional design, longitudinal and intervention studies are needed to clarify temporal relationships and evaluate potential approaches to addressing loneliness.

## Supplementary Information

Supplementary Material 1.

## Data Availability

The scientific use files of FReDA are online available at www.gesis.org: FReDA – The German Family Demography Panel Study. GESIS, Cologne. ZA7777 Data file Version 4.1.0, DOI: 10.4232/1.14398.
