# Supplementary material for: Overall, emotional and social loneliness and their associated factors: a large-scale cross-sectional study of young and middle-aged adults in Germany
Source: BMC Public Health. 2026 Oct 29;26:3109. doi: 10.1186/s12889-026-30122-4 (PMC13721316; doi:10.1186/s12889-026-30122-4)
Supplement: Supplementary file 1 [file 12889_2026_30122_MOESM1_ESM.docx]

**S u p p l e m e n t a r y	M a t e r i a l**

**Overall, emotional and social loneliness among young and middle-aged adults and their associated factors**

Pauline Kleinschlömer*^1^, Helena Ludwig-Walz*^1,2^, Sabine Diabaté^1^, Martin Bujard^1,2^

* *These authors contributed equally*

^1^ Federal Institute for Population Research (BiB), Germany

^2^ Institute of Medical Psychology, University Heidelberg, Heidelberg, Germany

Corresponding authors: [pauline.kleinschloemer@bib.bund.de](mailto:pauline.kleinschloemer@bib.bund.de); helena.ludwig-walz@bib.bund.de

Federal Institute for Population Research (BiB)

Friedrich-Ebert-Allee 4

65185 Wiesbaden, Germany

**Table A1 |** Distribution of overall loneliness measured on the original scale, ranging from 0 to 6, and adjusted scale, ranging from 0 to 3.

| Overall Loneliness | | | | | |
| --- | --- | --- | --- | --- | --- |
| Original Scale (0-6) | |  | Adjusted Scale (0-3) | |  |
|  | Mean: 3.05 |  |  | Mean: 1.52 |  |
| 0 (not lonely) | 11.9% |  | 0 (not lonely) | 11.9% |  |
| 1 | 17.0% |  | 0.5 | 17.0% |  |
| 2 | 14.3% |  | 1 | 14.3% |  |
| 3 | 14.6% |  | 1.5 | 14.6% |  |
| 4 | 12.7% |  | 2 | 12.7% |  |
| 5 | 12.8% |  | 2.5 | 12.8% |  |
| 6 (intensely lonely) | 16.8% |  | 3 (intensely lonely) | 16.8% |  |

*Source:* FReDA Panel, wave 1 (autumn/winter 2021/22). Authors’ own calculations.

**Table A2 |** Correlation matrix of variables included in the full model.

| Variables | (1) | (2) | (3) | (4) | (5) | (6) | (7) | (8) | (9) | (10) | (11) | (12) | (13) | (14) | (15) | (16) | (17) | (18) | (19) | (20) | (21) | (22) | (23) | (24) | (25) | (26) |
| --- | --- | --- | --- | --- | --- | --- | --- | --- | --- | --- | --- | --- | --- | --- | --- | --- | --- | --- | --- | --- | --- | --- | --- | --- | --- | --- |
| (1) Overall Loneliness | 1.00 |  |  |  |  |  |  |  |  |  |  |  |  |  |  |  |  |  |  |  |  |  |  |  |  |  |
| (2) Social Loneliness | 0.88 | 1.00 |  |  |  |  |  |  |  |  |  |  |  |  |  |  |  |  |  |  |  |  |  |  |  |  |
| (3) Emot. Loneliness | 0.83 | 0.47 | 1.00 |  |  |  |  |  |  |  |  |  |  |  |  |  |  |  |  |  |  |  |  |  |  |  |
| (4) Age | -0.00 | 0.08 | -0.10 | 1.00 |  |  |  |  |  |  |  |  |  |  |  |  |  |  |  |  |  |  |  |  |  |  |
| (5) Gender | 0.01 | -0.03 | 0.06 | -0.02 | 1.00 |  |  |  |  |  |  |  |  |  |  |  |  |  |  |  |  |  |  |  |  |  |
| (6) Financ. Satisfaction | -0.22 | -0.18 | -0.20 | 0.00 | -0.04 | 1.00 |  |  |  |  |  |  |  |  |  |  |  |  |  |  |  |  |  |  |  |  |
| (7) Edu. Background | -0.12 | -0.08 | -0.12 | 0.21 | -0.01 | 0.25 | 1.00 |  |  |  |  |  |  |  |  |  |  |  |  |  |  |  |  |  |  |  |
| (8) Migration Background | 0.05 | 0.05 | 0.03 | -0.02 | 0.00 | -0.07 | -0.05 | 1.00 |  |  |  |  |  |  |  |  |  |  |  |  |  |  |  |  |  |  |
| (9) Children | -0.03 | 0.03 | -0.09 | 0.59 | 0.07 | -0.06 | 0.11 | -0.03 | 1.00 |  |  |  |  |  |  |  |  |  |  |  |  |  |  |  |  |  |
| (10) Partnership Status | -0.13 | -0.07 | -0.16 | 0.33 | 0.06 | 0.10 | 0.21 | -0.03 | 0.47 | 1.00 |  |  |  |  |  |  |  |  |  |  |  |  |  |  |  |  |
| (11) Social Network | -0.10 | -0.15 | -0.01 | -0.08 | 0.11 | 0.04 | 0.06 | -0.00 | -0.12 | -0.17 | 1.00 |  |  |  |  |  |  |  |  |  |  |  |  |  |  |  |
| (12) Anxiety | 0.14 | 0.10 | 0.14 | 0.04 | 0.09 | -0.12 | -0.05 | -0.01 | -0.03 | -0.05 | 0.03 | 1.00 |  |  |  |  |  |  |  |  |  |  |  |  |  |  |
| (13) Depressive Mood | 0.23 | 0.15 | 0.24 | -0.12 | 0.07 | -0.18 | -0.12 | 0.05 | -0.12 | -0.15 | 0.00 | 0.24 | 1.00 |  |  |  |  |  |  |  |  |  |  |  |  |  |
| (14) Life Satisfaction | -0.35 | -0.27 | -0.34 | 0.01 | -0.00 | 0.28 | 0.10 | -0.01 | 0.09 | 0.20 | -0.00 | -0.23 | -0.36 | 1.00 |  |  |  |  |  |  |  |  |  |  |  |  |
| (15) COVID: positive | 0.18 | 0.08 | 0.24 | -0.02 | 0.13 | -0.18 | -0.05 | 0.02 | 0.03 | -0.03 | 0.04 | 0.09 | 0.17 | -0.23 | 1.00 |  |  |  |  |  |  |  |  |  |  |  |
| (16) COVID: negative | -0.13 | -0.10 | -0.13 | -0.03 | 0.04 | 0.13 | 0.06 | -0.01 | -0.04 | 0.03 | 0.04 | -0.01 | -0.07 | 0.18 | -0.35 | 1.00 |  |  |  |  |  |  |  |  |  |  |
| (17) Typ2 Diabetes | 0.02 | 0.02 | 0.01 | 0.09 | 0.02 | -0.04 | -0.02 | 0.01 | 0.04 | 0.01 | -0.00 | 0.04 | 0.02 | -0.04 | 0.01 | -0.00 | 1.00 |  |  |  |  |  |  |  |  |  |
| (18) Hyptertension | 0.04 | 0.06 | 0.01 | 0.19 | -0.09 | -0.05 | -0.02 | -0.01 | 0.08 | 0.02 | -0.04 | 0.06 | 0.02 | -0.08 | 0.01 | -0.02 | 0.13 | 1.00 |  |  |  |  |  |  |  |  |
| (19) Cancer | 0.02 | 0.02 | 0.01 | 0.08 | 0.03 | -0.02 | 0.00 | 0.00 | 0.04 | 0.01 | -0.01 | 0.04 | 0.02 | -0.04 | 0.02 | -0.00 | 0.04 | 0.02 | 1.00 |  |  |  |  |  |  |  |
| (20) Asthma | 0.04 | 0.03 | 0.04 | -0.02 | 0.00 | -0.05 | -0.02 | -0.00 | -0.02 | -0.02 | 0.01 | 0.06 | 0.04 | -0.05 | 0.03 | 0.01 | 0.02 | 0.02 | -0.00 | 1.00 |  |  |  |  |  |  |
| (21) Obesity | 0.06 | 0.07 | 0.03 | 0.12 | -0.01 | -0.13 | -0.09 | -0.00 | 0.06 | 0.01 | -0.03 | 0.04 | 0.04 | -0.07 | 0.02 | -0.01 | 0.14 | 0.25 | 0.01 | 0.04 | 1.00 |  |  |  |  |  |
| (22) Neuroticism | 0.21 | 0.12 | 0.25 | -0.14 | 0.09 | -0.16 | -0.09 | 0.03 | -0.12 | -0.11 | 0.01 | 0.18 | 0.28 | -0.29 | 0.18 | -0.05 | 0.01 | 0.01 | -0.00 | 0.05 | 0.04 | 1.00 |  |  |  |  |
| (23) Openess | -0.05 | -0.07 | -0.00 | -0.02 | -0.01 | 0.05 | 0.12 | 0.05 | -0.07 | -0.07 | 0.11 | 0.04 | 0.02 | 0.02 | 0.01 | 0.10 | -0.01 | -0.03 | 0.00 | 0.03 | -0.05 | 0.01 | 1.00 |  |  |  |
| (24) Agreeableness | -0.13 | -0.16 | -0.06 | -0.02 | 0.17 | 0.03 | 0.04 | 0.01 | 0.01 | 0.02 | 0.10 | -0.00 | -0.04 | 0.13 | 0.03 | 0.05 | 0.00 | -0.03 | -0.00 | 0.02 | -0.05 | 0.03 | 0.10 | 1.00 |  |  |
| (25) Consiousness | -0.12 | -0.08 | -0.12 | 0.12 | 0.09 | 0.08 | 0.05 | 0.01 | 0.13 | 0.12 | -0.05 | -0.08 | -0.11 | 0.16 | -0.02 | -0.00 | -0.01 | -0.02 | 0.03 | -0.03 | -0.09 | -0.13 | -0.01 | 0.11 | 1.00 |  |
| (26) Extraversion | -0.16 | -0.15 | -0.12 | 0.02 | 0.04 | 0.11 | 0.09 | -0.01 | 0.07 | 0.11 | 0.08 | -0.10 | -0.14 | 0.22 | 0.00 | 0.04 | -0.01 | -0.04 | 0.00 | -0.01 | -0.03 | -0.25 | 0.18 | 0.04 | 0.21 | 1.00 |
| *Source:* FReDA Panel, wave 1 (autumn/winter 2021/22). Authors’ own calculations. | | | | | | | | | | | | | | | | | | | | | | | | | | |

**Table A3 |** Item Battery (Big Five Inventory – 2 Extra Short Form)

| Extraversion | I tend to be quiet. (R) |
| --- | --- |
|  | I am dominant, act as a lead. |
|  | I am full of energy. |
| Agreeableness | I am compassionate, have a soft heart |
|  | I am sometimes rude to others. (R) |
|  | I assume the best about people. |
| Conscientiousness | I tend to be disorganised. (R) |
|  | I have difficulty getting started on tasks. (R) |
|  | I am reliable, can always be counted on. |
| Neuroticism | I worry a lot. |
|  | I tend to feel depressed, blue. |
|  | I am emotionally stable, not easily upset. (R) |
| Openness to new experiences | I am fascinated by art, music, or literature. |
|  | I have little interest in abstract ideas. (R) |
|  | I am original, come up with ideas. |
| *Reversed-keyed items are denoted by “R”. | |

*Source:* Soto, C. J., & John, O. P. (2017). Short and extra-short forms of the Big Five Inventory–2: The BFI-2-S and BFI-2-XS. Journal of Research in Personality, 68, 69-81.

**Table A4 |** Comparison of complete case analysis and multiple imputation (overall loneliness)

|  |  | Overall Loneliness  (complete case analysis) | | | | | Overall Loneliness (multiple imputation) | | | | |
| --- | --- | --- | --- | --- | --- | --- | --- | --- | --- | --- | --- |
|  |  | ß Coef. | p-value | | 95 % CI | | ß Coef. | p-value | | 95 % CI | |
| Base Model | Age : |  |  | |  |  |  |  | |  |  |
|  | Ref.: 18-25 | 0 | . | | . | . | 0 | . | | . | . |
|  | 26-30 | .001 | .963 | | -.052 | .054 | .003 | .914 | | -.047 | 0.053 |
|  | 31-35 | .06 | .029 | | .006 | .115 | .070 | .007 | | .020 | 0.121 |
|  | 36-40 | .061 | .038 | | .003 | .119 | .071 | .011 | | .016 | 0.125 |
|  | 41-45 | .076 | .015 | | .015 | .136 | .073 | .014 | | .015 | 0.130 |
|  | 46-51 | .09 | .005 | | .027 | .154 | .090 | .003 | | .030 | 0.149 |
|  | Sex |  |  | |  |  |  |  | |  |  |
|  | Ref.: Male | 0 | . | | . | . | 0 | . | | . | . |
|  | Female | .041 | .009 | | .01 | .072 | .025 | .089 | | -.004 | .055 |
| Socio-Demographic Variables | Satisfaction with HH-Income | -.064 | 0 | | -.078 | -.051 | -.073 | 0 | | -.086 | -.061 |
|  | Educational Background |  |  | |  |  |  |  | |  |  |
|  | Ref.: low | 0 | . | | . | . | 0 | . | | . | . |
|  | middle | .002 | .96 | | -.088 | .092 | -.028 | .497 | | -.108 | .053 |
|  | high | -.079 | .089 | | -.17 | .012 | -.114 | .006 | | -.195 | -.032 |
|  | Migration Background |  |  | |  |  |  |  | |  |  |
|  | Ref.: No | 0 | . | | . | . | 0 | . | | . | . |
|  | 1. Generation | .207 | 0 | | .154 | .26 | 0.219 | 0 | | .171 | .267 |
|  | 2. Generation | .047 | .049 | | 0 | .094 | 0.052 | .020 | | .008 | .096 |
| Social Relationships | Children |  |  | |  |  |  |  | |  |  |
|  | Ref.: No | 0 | . | | . | . | 0 | . | | . | . |
|  | Yes | .006 | .755 | | -.034 | .047 | 0.0005 | .980 | | -.039 | .038 |
|  | Partnership Status |  |  | |  |  |  |  | |  |  |
|  | Ref.: Single | 0 | . | | . | . | 0 | . | | . | . |
|  | LAT | -.182 | 0 | | -.234 | -.13 | -.180 | 0 | | -.229 | -.131 |
|  | Cohabitation | -.184 | 0 | | -.226 | -.143 | -.175 | 0 | | -.215 | -.136 |
|  | Social Network |  |  | |  |  |  |  | |  |  |
|  | Ref.: No | 0 | . | | . | . | 0 | . | | . | . |
|  | Yes | -.195 | 0 | | -.226 | -.163 | -.183 | 0 | | -.212 | -.153 |
| Health Status | Anxiety Disorder |  |  | |  |  |  |  | |  |  |
|  | Ref.: No | 0 | . | | . | . | 0 | . | | . | . |
|  | Yes | .107 | 0 | | .057 | .158 | .108 | 0 | | .060 | .156 |
|  | Depressive Mood |  |  | |  |  |  |  | |  |  |
|  | Ref.: No | 0 | . | | . | . | 0 | . | | . | . |
|  | Yes | .189 | 0 | | .14 | .238 | .186 | 0 | | .140 | .232 |
|  | Life Satisfaction | -.117 | 0 | | -.127 | -.108 | -.119 | 0 | | -.128 | -.110 |
|  | Covid19: Positive Aspects | -.029 | 0 | | -.043 | -.015 | -.027 | 0 | | -.041 | -.014 |
|  | Covid19: Personal Burden | .069 | 0 | | .055 | .083 | .069 | 0 | | .055 | .082 |
|  | Typ 2 Diabetes |  |  | |  |  |  |  | |  |  |
|  | Ref.: No | 0 | . | | . | . | 0 | . | | . | . |
|  | Yes | -.054 | .27 | | -.15 | .042 | -.011 | 0.803 | | -.101 | .078 |
|  | Hypertonie |  |  | |  |  |  |  | |  |  |
|  | Ref.: No | 0 | . | | . | . | 0 | . | | . | . |
|  | Yes | .016 | .545 | | -.036 | .068 | .029 | 0.251 | | -.020 | .078 |
|  | Cancer |  |  | |  |  |  |  | |  |  |
|  | Ref.: No | 0 | . | | . | . | 0 | . | | . | . |
|  | Yes | .031 | .569 | | -.076 | .138 | .035 | 0.495 | | -.065 | .135 |
|  | Asthma |  |  | |  |  |  |  | |  |  |
|  | Ref.: No | 0 | . | | . | . | 0 | . | | . | . |
|  | Yes | .049 | .059 | | -.002 | .101 | .036 | 0.148 | | -.013 | .084 |
|  | Obesity |  |  | |  |  |  |  | |  |  |
|  | Ref.: No | 0 | . | | . | . | 0 | . | | . | . |
|  | Yes | .003 | .903 | | -.04 | .045 | .005 | 0.798 | | -.035 | .045 |
| Big Five | Neuroticism | .121 | 0 | | .096 | .147 | .101 | 0 | | .075 | .127 |
|  | Openness for Experience | -.017 | .095 | | -.036 | .003 | -.014 | 0.203 | | -.035 | .007 |
|  | Agreeableness | -.119 | 0 | | -.14 | -.098 | -.100 | 0 | | -.121 | -.079 |
|  | Conscientiousness | -.053 | 0 | | -.073 | -.033 | -.043 | 0 | | -.064 | -.023 |
|  | Extraversion | -.052 | 0 | | -.073 | -.03 | -.042 | 0 | | -.064 | -.021 |
|  | Constant | 3.121 | 0 | | 2.925 | 3.319 | 3.130 | 0 | | 2.932 | 3.326 |
|  | Number of obs | 14,436 | | | | | 16,771 | | | | |
|  | R-squared | | | 0.198 | | | | |  |  |  |

*Source:* FReDA Panel, wave 1 (autumn/winter 2021/22). Authors’ own calculations.

**Table A5 |** Comparison of complete case analysis and multiple imputation (emotional loneliness)

|  |  | Emotional Loneliness  (complete case analysis) | | | | Emotional Loneliness (multiple imputation) | | | | |
| --- | --- | --- | --- | --- | --- | --- | --- | --- | --- | --- |
|  |  | Coef. | p-value | 95 % CI | | Coef. | | p-value | 95 % CI | |
| Base Model | Age : |  |  |  |  |  | |  |  |  |
|  | Ref.: 18-25 | 0 | . | . | . | 0 | | . | . | . |
|  | 26-30 | -.032 | .275 | -.09 | .026 | -.028 | | .310 | -.082 | .026 |
|  | 31-35 | -.056 | .064 | -.115 | .003 | -.042 | | .136 | -.097 | .013 |
|  | 36-40 | -.122 | 0 | -.185 | -.059 | -.111 | | 0 | -.170 | -.052 |
|  | 41-45 | -.142 | 0 | -.208 | -.076 | -.145 | | 0 | -.207 | -.083 |
|  | 46-51 | -.139 | 0 | -.208 | -.071 | -.145 | | 0 | -.209 | -.081 |
|  | Sex |  |  |  |  |  | |  |  |  |
|  | Ref.: Male | 0 | . | . | . | 0 | | . | . | . |
|  | Female | .086 | 0 | .052 | .12 | .077 | | 0 | .046 | .109 |
| Socio-Demographic Variables | Satisfaction with HH-Income | -.058 | 0 | -.072 | -.043 | -.067 | | 0 | -.081 | -.053 |
|  | Educational Background |  |  |  |  |  | |  |  |  |
|  | Ref.: low | 0 | . | . | . | 0 | | . | . | . |
|  | middle | .049 | .326 | -.049 | .147 | -.007 | | .878 | -.094 | .080 |
|  | high | -.034 | .5 | -.133 | .065 | -.087 | | .052 | -.176 | .001 |
|  | Migration Background |  |  |  |  |  | |  |  |  |
|  | Ref.: No | 0 | . | . | . | 0 | | . | . | . |
|  | 1. Generation | .103 | 0 | .045 | .16 | .129 | | 0 | .077 | .181 |
|  | 2. Generation | .019 | .457 | -.032 | .07 | .016 | | .508 | -.032 | .064 |
| Social Relationships | Children |  |  |  |  |  | |  |  |  |
|  | Ref.: No | 0 | . | . | . | 0 | | . | . | . |
|  | Yes | -.005 | .82 | -.049 | .039 | -.019 | | .377 | -.060 | .023 |
|  | Partnership Status |  |  |  |  |  | |  |  |  |
|  | Ref.: Single | 0 | . | . | . | 0 | | . | . | . |
|  | LAT | -.137 | 0 | -.194 | -.08 | -.136 | | 0 | -.189 | -.084 |
|  | Cohabitation | -.16 | 0 | -.205 | -.115 | -.148 | | 0 | -.191 | -.106 |
|  | Social Network |  |  |  |  |  | |  |  |  |
|  | Ref.: No | 0 | . | . | . | 0 | | . | . | . |
|  | Yes | -.066 | 0 | -.1 | -.031 | -.053 | | .001 | -.085 | -.021 |
| Health Status | Anxiety Disorder |  |  |  |  |  | |  |  |  |
|  | Ref.: No | 0 | . | . | . | 0 | | . | . | . |
|  | Yes | .113 | 0 | .058 | .167 | .114 | | 0 | .062 | .166 |
|  | Depressive Mood |  |  |  |  |  | |  |  |  |
|  | Ref.: No | 0 | . | . | . | 0 | | . | . | . |
|  | Yes | .218 | 0 | .165 | .271 | .222 | | 0 | .172 | .271 |
|  | Life Satisfaction | -.116 | 0 | -.126 | -.105 |  | |  |  |  |
|  | Covid19: Positive Aspects | -.023 | .004 | -.039 | -.007 | -.117 | | 0 | -.127 | -.108 |
|  | Covid19: Personal Burden | .125 | 0 | .11 | .141 | -.023 | | .002 | -.037 | -.008 |
|  | Typ 2 Diabetes |  |  |  |  | .125 | | 0 | .110 | .139 |
|  | Ref.: No | 0 | . | . | . | 0 | | . | . | . |
|  | Yes | -.056 | .286 | -.16 | .047 | -.014 | | .784 | -.111 | .083 |
|  | Hypertonie |  |  |  |  |  | |  |  |  |
|  | Ref.: No | 0 | . | . | . | 0 | | . | . | . |
|  | Yes | -.011 | .707 | -.067 | .045 | .003 | | .902 | -.050 | .057 |
|  | Cancer |  |  |  |  |  | |  |  |  |
|  | Ref.: No | 0 | . | . | . | 0 | | . | . | . |
|  | Yes | .041 | .489 | -.075 | .157 | .051 | | .357 | -.058 | .159 |
|  | Asthma |  |  |  |  |  | |  |  |  |
|  | Ref.: No | 0 | . | . | . | 0 | | . | . | . |
|  | Yes | .042 | .139 | -.014 | .098 | .032 | | .229 | -.020 | .085 |
|  | Obesity |  |  |  |  |  | |  |  |  |
|  | Ref.: No | 0 | . | . | . | 0 | | . | . | . |
|  | Yes | -.018 | .435 | -.064 | .028 | -.011 | | .621 | -.054 | .032 |
| Big Five | Neuroticism | .171 | 0 | .144 | .199 | .143 | | 0 | .114 | .171 |
|  | Openness for Experience | .01 | .369 | -.011 | .031 | .008 | | .465 | -.014 | .031 |
|  | Agreeableness | -.045 | 0 | -.068 | -.022 | -.037 | | .001 | -.060 | -.014 |
|  | Conscientiousness | -.065 | 0 | -.086 | -.043 | -.053 | | 0 | -.075 | -.032 |
|  | Extraversion | -.022 | .069 | -.045 | .002 | -.016 | | .171 | -.039 | .007 |
|  |  |  |  |  |  |  | |  |  |  |
|  | Constant | 2.286 | 0 | 2.073 | 2.499 | 2.401 | | 0 | 2.190 | 2.613 |
|  | Number of obs | 14,436 | | | | 16,771 | | | | |
|  | R-squared | 0.191 | | | | |  | | | |

*Source:* FReDA Panel, wave 1 (autumn/winter 2021/22). Authors’ own calculations.

**Table A6 |** Comparison of complete case analysis and multiple imputation (social loneliness)

|  |  | Social Loneliness  (complete case analysis) | | | | Social Loneliness (multiple imputation) | | | |
| --- | --- | --- | --- | --- | --- | --- | --- | --- | --- |
|  |  | Coef. | p-value | 95 % CI | | Coef. | p-value | 95 % CI | |
| Base Model | Age : |  |  |  |  |  |  |  |  |
|  | Ref.: 18-25 | 0 | . | . | . | 0 | . | . | . |
|  | 26-30 | .035 | .323 | -.034 | .103 | .033 | .305 | -.031 | .097 |
|  | 31-35 | .177 | 0 | .107 | .247 | .182 | 0 | .117 | .248 |
|  | 36-40 | .244 | 0 | .169 | .319 | .253 | 0 | .183 | .32 |
|  | 41-45 | .293 | 0 | .214 | .372 | .289 | 0 | .215 | .362 |
|  | 46-51 | .32 | 0 | .238 | .402 | .324 | 0 | .248 | .400 |
|  | Sex |  |  |  |  |  |  |  |  |
|  | Ref.: Male | 0 | . | . | . | 0 | . | . | . |
|  | Female | -.004 | .855 | -.044 | .036 | -.026 | 0.171 | -.064 | .011 |
| Socio-Demographic Variables | Satisfaction with HH-Income | -.071 | 0 | -.089 | -.054 | -.080 | 00 | -.096 | -.063 |
|  | Educational Background |  |  |  |  |  |  |  |  |
|  | Ref.: low | 0 | . | . | . | 0 | . | . | . |
|  | middle | -.044 | .454 | -.161 | .072 | -.049 | 0.352 | -.152 | .054 |
|  | high | -.124 | .039 | -.242 | -.006 | -.140 | 0.01 | -.244 | -.035 |
|  | Migration Background |  |  |  |  |  |  |  |  |
|  | Ref.: No | 0 | . | . | . | 0 | . | . | . |
|  | 1. Generation | .311 | 0 | .242 | .379 | .309 | 0 | .247 | .371 |
|  | 2. Generation | .075 | .015 | .014 | .136 | .088 | 0.002 | .032 | .145 |
| Social Relationships | Children |  |  |  |  |  |  |  |  |
|  | Ref.: No | 0 | . | . | . | 0 | . | . | . |
|  | Yes | .018 | .5 | -.034 | .07 | .018 | 0.481 | -.031 | .067 |
|  | Partnership Status |  |  |  |  |  |  |  |  |
|  | Ref.: Single | 0 | . | . | . | 0 | . | . | . |
|  | LAT | -.227 | 0 | -.294 | -.16 | -.224 | 0 | -.287 | -.162 |
|  | Cohabitation | -.208 | 0 | -.262 | -.154 | -.203 | 0 | -.253 | -.152 |
|  | Social Network |  |  |  |  |  |  |  |  |
|  | Ref.: No | 0 | . | . | . | 0 | . | . | . |
|  | Yes | -.324 | 0 | -.365 | -.283 | -.312 | 0 | -.350 | -.274 |
| Health Status | Anxiety Disorder |  |  |  |  |  |  |  |  |
|  | Ref.: No | 0 | . | . | . | 0 | . | . | . |
|  | Yes | .102 | .002 | .037 | .168 | .102 | 0.001 | .041 | .164 |
|  | Depressive Mood |  |  |  |  |  |  |  |  |
|  | Ref.: No | 0 | . | . | . | 0 | . | . | . |
|  | Yes | .16 | 0 | .097 | .223 | .149 | 0 | .090 | .208 |
|  | Life Satisfaction | -.118 | 0 | -.131 | -.106 | -.121 | 0 | -.133 | -.110 |
|  | Covid19: Positive Aspects | -.035 | 0 | -.054 | -.017 | -.032 | 0 | -.049 | -.015 |
|  | Covid19: Personal Burden | .012 | .184 | -.006 | .031 | .013 | 0.143 | -.004 | .030 |
|  | Typ 2 Diabetes |  |  |  |  |  |  |  |  |
|  | Ref.: No | 0 | . | . | . | 0 | . | . | . |
|  | Yes | -.051 | .416 | -.175 | .072 | -.009 | 0.875 | -.124 | .106 |
|  | Hypertonie |  |  |  |  |  |  |  |  |
|  | Ref.: No | 0 | . | . | . | 0 | . | . | . |
|  | Yes | .043 | .21 | -.024 | .11 | .054 | 0.091 | -.009 | .118 |
|  | Cancer |  |  |  |  |  |  |  |  |
|  | Ref.: No | 0 | . | . | . | 0 | . | . | . |
|  | Yes | .021 | .764 | -.117 | .159 | .019 | 0.773 | -.110 | .148 |
|  | Asthma |  |  |  |  |  |  |  |  |
|  | Ref.: No | 0 | . | . | . | 0 | . | . | . |
|  | Yes | .057 | .092 | -.009 | .123 | .039 | 0.214 | -.023 | .102 |
|  | Obesity |  |  |  |  |  |  |  |  |
|  | Ref.: No | 0 | . | . | . | 0 | . | . | . |
|  | Yes | .024 | .398 | -.031 | .078 | .021 | 0.415 | -.030 | .072 |
| Big Five | Neuroticism | .071 | 0 | .038 | .104 | .059 | 0.001 | .026 | .092 |
|  | Openness for Experience | -.043 | .001 | -.068 | -.018 | -.036 | 0.009 | -.062 | -.009 |
|  | Agreeableness | -.193 | 0 | -.22 | -.166 | -.163 | 0 | -.190 | -.136 |
|  | Conscientiousness | -.042 | .002 | -.068 | -.016 | -.034 | 0.011 | -.060 | -.008 |
|  | Extraversion | -.082 | 0 | -.11 | -.054 | -.068 | 0 | -.096 | -.041 |
|  |  |  |  |  |  |  |  |  |  |
|  | Constant | 3.958 | 0 | 3.704 | 4.212 | 3.858 | 0 | 3.607 | 4.109 |
|  |  |  |  |  |  |  |  |  |  |
|  | Number of obs | 14.436 | | | | 16.771 | | | |
|  | R-squared | 0.15 | | | |  | | | |

*Source:* FReDA Panel, wave 1 (autumn/winter 2021/22). Authors’ own calculations.

**Table A7 |** Regression Tables. Base Model for Overall Loneliness, Emotional Loneliness and Social Loneliness

| *Overall Loneliness* |  |  |  | |
| --- | --- | --- | --- | --- |
| Age | Predicted Value | p-value | 95% Confidence Interval | |
| 18-25 | 1.497 | 0.000 | 1.460 | 1.534 |
| 26-30 | 1.382 | 0.000 | 1.339 | 1.423 |
| 31-35 | 1.411 | 0.000 | 1.373 | 1.449 |
| 36-40 | 1.425 | 0.000 | 1.386 | 1.467 |
| 41-45 | 1.449 | 0.000 | 1.407 | 1.491 |
| 46-51 | 1.466 | 0.000 | 1.422 | 1.510 |
| *Emotional Loneliness* |  |  |  | |
| Age | Predicted Value | p-value | 95% Confidence Interval | |
| 18-25 | 1.650 | 0.000 | 1.610 | 1.689 |
| 26-30 | 1.479 | 0.000 | 1.434 | 1.524 |
| 31-35 | 1.402 | 0.000 | 1.362 | 1.443 |
| 36-40 | 1.356 | 0.000 | 1.304 | 1.387 |
| 41-45 | 1.331 | 0.000 | 1.286 | 1.375 |
| 46-51 | 1.328 | 0.000 | 1.281 | 1.376 |
| *Social Loneliness* |  |  |  | |
| Age | Predicted Value | p-value | 95% Confidence Interval | |
| 18-25 | 1.345 | 0.000 | 1.301 | 1.391 |
| 26-30 | 1.284 | 0.000 | 1.232 | 1.337 |
| 31-35 | 1.420 | 0.000 | 1.373 | 1.468 |
| 36-40 | 1.504 | 0.000 | 1.456 | 1.553 |
| 41-45 | 1.567 | 0.000 | 1.514 | 1.619 |
| 46-51 | 1.603 | 0.000 | 1.548 | 1.658 |

Note: Estimated predicted values are reported at the mean values of all covariates, meaning that the coefficents represent the expected outcome when all independent variables are set to their sample means within each age group.

Source: FReDA Panel, wave 1 (autumn/winter 2021/22). Authors’ own calculations.

**Table A8 |** Stepwise Regressiontable (Overall Loneliness)

|  |  | Base Model | | | | Model 1 | | | | Model 2 | | | | Model 3 | | | | Model 4 | | | |
| --- | --- | --- | --- | --- | --- | --- | --- | --- | --- | --- | --- | --- | --- | --- | --- | --- | --- | --- | --- | --- | --- |
|  |  | Coef. | p-value | 95 % CI | | Coef. | p-value | 95 % CI | | Coef. | p-value | 95 % CI | | Coef. | p-value | 95 % CI | | Coef. | p-value | 95 % CI | |
| Base Model | Age : |  |  |  |  |  |  |  |  |  |  |  |  |  |  |  |  |  |  |  |  |
|  | Ref.: 18-25 | 0 | . | . | . | 0 | . | . | . | 0 | . | . | . | 0 | . | . | . | 0 | . | . | . |
|  | 26-30 | -.116 | 0 | -.172 | -.060 | -.061 | .03 | -.117 | -.006 | .006 | .825 | -.05 | .063 | -.005 | .868 | -.06 | .05 | .001 | .963 | -.052 | .054 |
|  | 31-35 | -.086 | .001 | -.139 | -.033 | -.035 | .197 | -.087 | .018 | .075 | .01 | .018 | .132 | .052 | .07 | -.004 | .108 | .06 | .029 | .006 | .115 |
|  | 36-40 | -.072 | .01 | -.126 | -.019 | -.033 | .217 | -.086 | .02 | .093 | .003 | .032 | .154 | .058 | .058 | -.002 | .117 | .061 | .038 | .003 | .119 |
|  | 41-45 | -.049 | .09 | -.104 | .007 | -.011 | .695 | -.066 | .044 | .117 | 0 | .054 | .181 | .061 | .056 | -.002 | .123 | .076 | .015 | .015 | .136 |
|  | 46-51 | -.032 | .281 | -.089 | .026 | -.004 | .886 | -.061 | .052 | .119 | 0 | .053 | .184 | .064 | .053 | -.001 | .129 | .09 | .005 | .027 | .154 |
|  | Sex |  |  |  |  |  |  |  |  |  |  |  |  |  |  |  |  |  |  |  |  |
|  | Ref.: Male | 0 | . | . | . | 0 | . | . | . | 0 | . | . | . | 0 | . | . | . | 0 | . | . | . |
|  | Female | .024 | .158 | -.009 | .057 | .005 | .752 | -.027 | .037 | .054 | .001 | .022 | .086 | .014 | .374 | -.017 | .045 | .041 | .009 | .01 | .072 |
| Socio-Demographic Variables | Satisfaction with HH-Income |  |  |  |  | -.167 | 0 | -.18 | -.153 | -.157 | 0 | -.171 | -.143 | -.071 | 0 | -.084 | -.057 | -.064 | 0 | -.078 | -.051 |
|  | Educational Background |  |  |  |  |  |  |  |  |  |  |  |  |  |  |  |  |  |  |  |  |
|  | Ref.: low | 0 | . | . | . | 0 | . | . | . | 0 | . | . | . | 0 | . | . | . | 0 | . | . | . |
|  | middle |  |  |  |  | -.084 | .091 | -.181 | .013 | -.038 | .44 | -.134 | .058 | -.008 | .828 | -.084 | .067 | .002 | .96 | -.088 | .092 |
|  | high |  |  |  |  | -.219 | 0 | -.317 | -.121 | -.148 | .003 | -.245 | -.051 | -.085 | .035 | -.164 | -.006 | -.079 | .089 | -.17 | .012 |
|  | Migration Background |  |  |  |  |  |  |  |  |  |  |  |  |  |  |  |  |  |  |  |  |
|  | Ref.: No | 0 | . | . | . | 0 | . | . | . | 0 | . | . | . | 0 | . | . | . | 0 | . | . | . |
|  | 1. Generation |  |  |  |  | .131 | 0 | .074 | .188 | .128 | 0 | .072 | .185 | .2 | 0 | .146 | .253 | .207 | 0 | .154 | .26 |
|  | 2. Generation |  |  |  |  | .061 | .019 | .01 | .111 | .059 | .022 | .009 | .109 | .046 | .057 | -.001 | .093 | .047 | .049 | 0 | .094 |
| Social Relationships | Children |  |  |  |  |  |  |  |  |  |  |  |  |  |  |  |  |  |  |  |  |
|  | Ref.: No |  |  |  |  |  |  |  |  | 0 | . | . | . | 0 | . | . | . | 0 | . | . | . |
|  | Yes |  |  |  |  |  |  |  |  | -.059 | .007 | -.102 | -.016 | -.003 | .881 | -.044 | .038 | .006 | .755 | -.034 | .047 |
|  | Partnership Status |  |  |  |  |  |  |  |  |  |  |  |  |  |  |  |  |  |  |  |  |
|  | Ref.: Single |  |  |  |  |  |  |  |  | 0 | . | . | . | 0 | . | . | . | 0 | . | . | . |
|  | LAT |  |  |  |  |  |  |  |  | -.293 | 0 | -.349 | -.238 | -.192 | 0 | -.245 | -.14 | -.182 | 0 | -.234 | -.13 |
|  | Cohabitation |  |  |  |  |  |  |  |  | -.316 | 0 | -.36 | -.272 | -.193 | 0 | -.235 | -.15 | -.184 | 0 | -.226 | -.143 |
|  | Social Network |  |  |  |  |  |  |  |  |  |  |  |  |  |  |  |  |  |  |  |  |
|  | Ref.: No |  |  |  |  |  |  |  |  | 0 | . | . | . | 0 | . | . | . | 0 | . | . | . |
|  | Yes |  |  |  |  |  |  |  |  | -.225 | 0 | -.258 | -.191 | -.218 | 0 | -.249 | -.186 | -.195 | 0 | -.226 | -.163 |
| Health Status | Anxiety Disorder |  |  |  |  |  |  |  |  |  |  |  |  |  |  |  |  |  |  |  |  |
|  | Ref.: No |  |  |  |  |  |  |  |  |  |  |  |  | 0 | . | . | . | 0 | . | . | . |
|  | Yes |  |  |  |  |  |  |  |  |  |  |  |  | .135 | 0 | .084 | .186 | .107 | 0 | .057 | .158 |
|  | Depressive Mood |  |  |  |  |  |  |  |  |  |  |  |  |  |  |  |  |  |  |  |  |
|  | Ref.: No |  |  |  |  |  |  |  |  |  |  |  |  | 0 | . | . | . | 0 | . | . | . |
|  | Yes |  |  |  |  |  |  |  |  |  |  |  |  | .239 | 0 | .19 | .288 | .189 | 0 | .14 | .238 |
|  | Life Satisfaction |  |  |  |  |  |  |  |  |  |  |  |  | -.137 | 0 | -.146 | -.128 | -.117 | 0 | -.127 | -.108 |
|  | Covid19: Positive Aspects |  |  |  |  |  |  |  |  |  |  |  |  | -.03 | 0 | -.044 | -.015 | -.029 | 0 | -.043 | -.015 |
|  | Covid19: Personal Burden |  |  |  |  |  |  |  |  |  |  |  |  | .069 | 0 | .054 | .083 | .069 | 0 | .055 | .083 |
|  | Typ 2 Diabetes |  |  |  |  |  |  |  |  |  |  |  |  |  |  |  |  |  |  |  |  |
|  | Ref.: No |  |  |  |  |  |  |  |  |  |  |  |  | 0 | . | . | . | 0 | . | . | . |
|  | Yes |  |  |  |  |  |  |  |  |  |  |  |  | -.055 | .267 | -.152 | .042 | -.054 | .27 | -.15 | .042 |
|  | Hypertonie |  |  |  |  |  |  |  |  |  |  |  |  |  |  |  |  |  |  |  |  |
|  | Ref.: No |  |  |  |  |  |  |  |  |  |  |  |  | 0 | . | . | . | 0 | . | . | . |
|  | Yes |  |  |  |  |  |  |  |  |  |  |  |  | .018 | .504 | -.035 | .07 | .016 | .545 | -.036 | .068 |
|  | Cancer |  |  |  |  |  |  |  |  |  |  |  |  |  |  |  |  |  |  |  |  |
|  | Ref.: No |  |  |  |  |  |  |  |  |  |  |  |  | 0 | . | . | . | 0 | . | . | . |
|  | Yes |  |  |  |  |  |  |  |  |  |  |  |  | .013 | .811 | -.095 | .121 | .031 | .569 | -.076 | .138 |
|  | Asthma |  |  |  |  |  |  |  |  |  |  |  |  |  |  |  |  |  |  |  |  |
|  | Ref.: No |  |  |  |  |  |  |  |  |  |  |  |  | 0 | . | . | . | 0 | . | . | . |
|  | Yes |  |  |  |  |  |  |  |  |  |  |  |  | .045 | .087 | -.007 | .097 | .049 | .059 | -.002 | .101 |
|  | Obesity |  |  |  |  |  |  |  |  |  |  |  |  |  |  |  |  |  |  |  |  |
|  | Ref.: No |  |  |  |  |  |  |  |  |  |  |  |  | 0 | . | . | . | 0 | . | . | . |
|  | Yes |  |  |  |  |  |  |  |  |  |  |  |  | .028 | .191 | -.014 | .071 | .003 | .903 | -.04 | .045 |
| Big Five | Neuroticism |  |  |  |  |  |  |  |  |  |  |  |  |  |  |  |  | .121 | 0 | .096 | .147 |
|  | Openness for Experience |  |  |  |  |  |  |  |  |  |  |  |  |  |  |  |  | -.017 | .095 | -.036 | .003 |
|  | Agreeableness |  |  |  |  |  |  |  |  |  |  |  |  |  |  |  |  | -.119 | 0 | -.14 | -.098 |
|  | Conscientiousness |  |  |  |  |  |  |  |  |  |  |  |  |  |  |  |  | -.053 | 0 | -.073 | -.033 |
|  | Extraversion |  |  |  |  |  |  |  |  |  |  |  |  |  |  |  |  | -.052 | 0 | -.073 | -.03 |
|  |  |  |  |  |  |  |  |  |  |  |  |  |  |  |  |  |  |  |  |  |  |
|  | Constant | 1.484 | 0 | 1.443 | 1.525 | 2.36 | 0.00 | 2.248 | 2.473 | 2.551 | 0 | 2.437 | 2.664 | 2.853 | 0 | 2.706 | 3.00 | 3.122 | 0 | 2.925 | 3.319 |
|  |  |  |  |  |  |  |  |  |  |  |  |  |  | 0 | . | . | . |  |  |  |  |
|  | Number of obs | 14,443 | | | | 14,443 | | | | 14,443 | | | | 14,443 | | | | 14,443 | | | |
|  | R-squared | 0.002 | | | | 0.056 | | | | 0.081 | | | | 0.181 | | | | 0.199 | | | |

*Note:* In model 1 we control for socio-demographic variables (education, satisfaction with household income, migrant background). In model 2 we also control for social relationships, namely partnership status, children and discuss personal matters with social network (with friends, acquaintances, neighbours or colleagues). In model 3, we also included health status, including dimensions of physical health, mental health and COVID 19 variables. The full model, model 4, also includes the Big Five personality variables.

*Source:* FReDA panel, waves 1 (autumn/winter 2021/22). Authors’ own calculations.

**Table A9 |** Stepwise Regressiontable (Emotional Loneliness)

|  |  | Base Model | | | | Model 1 | | | | Model 2 | | | | Model 3 | | | | Model 4 | | | |
| --- | --- | --- | --- | --- | --- | --- | --- | --- | --- | --- | --- | --- | --- | --- | --- | --- | --- | --- | --- | --- | --- |
|  |  | Coef. | p- value | 95 %CI | | Coef. | p- value | 95 %CI | | Coef. | p- value | 95 %CI | | Coef. | p- value | 95 %CI | | Coef. | p- value | 95 %CI | |
|  | Age |  |  |  |  |  |  |  |  |  |  |  |  |  |  |  |  |  |  |  |  |
|  | Ref.: 18-25 | 0 | . | . | . | 0 | . | . | . | 0 | . | . | . | 0 | . | . | . | 0 | . | . | . |
|  | 26-30 | -.171 | 0 | -.231 | -.111 | -.126 | 0 | -.185 | -.066 | -.048 | .129 | -.109 | .014 | -.049 | .095 | -.107 | .009 | -.032 | .275 | -.09 | .026 |
|  | 31-35 | -.247 | 0 | -.304 | -.19 | -.201 | 0 | -.258 | -.144 | -.076 | .017 | -.138 | -.013 | -.08 | .008 | -.139 | -.021 | -.056 | .064 | -.115 | .003 |
|  | 36-40 | -.304 | 0 | -.361 | -.246 | -.269 | 0 | -.327 | -.212 | -.124 | 0 | -.19 | -.058 | -.155 | 0 | -.218 | -0.92 | -.122 | 0 | -.185 | -.059 |
|  | 41-45 | -.319 | 0 | -.379 | -.259 | -.285 | 0 | -.345 | -.226 | -.137 | 0 | -.206 | -.068 | -.185 | 0 | -.251 | -.119 | -.142 | 0 | -.208 | -.076 |
|  | 46-51 | -.321 | 0 | -.383 | -.259 | -.297 | 0 | -.358 | -.236 | -.148 | 0 | -.219 | -.077 | -.185 | 0 | -.254 | -.117 | -.139 | 0 | -.208 | -.071 |
|  | Sex |  |  |  |  |  |  |  |  |  |  |  |  |  |  |  |  |  |  |  |  |
|  | Ref.: Male | 0 | . | . | . | 0 | . | . | . | 0 | . | . | . | 0 | . | . | . | 0 | . | . | . |
|  | Female | .126 | 0 | .09 | .161 | .108 | 0 | .073 | .142 | .139 | 0 | .104 | .174 | .077 | 0 | .044 | .11 | .086 | 0 | .052 | .12 |
| Socio-Demographic Variables | Satisfaction with HH-Income |  |  |  |  | -.168 | 0 | -.183 | -.153 | -.161 | 0 | -.176 | -.146 | -.065 | 0 | -.079 | -.05 | -.058 | 0 | -.072 | -.043 |
|  | Educational Background |  |  |  |  |  |  |  |  |  |  |  |  |  |  |  |  |  |  |  |  |
|  | Ref.: low |  |  |  |  | 0 | . | . | . | 0 | . | . | . | 0 | . | . | . | 0 | . | . | . |
|  | middle |  |  |  |  | -.018 | .737 | -.123 | .087 | .011 | .837 | -.093 | .115 | .055 | .273 | -.043 | .153 | .049 | .326 | -.049 | .147 |
|  | high |  |  |  |  | -.126 | .019 | -.232 | -.02 | -.085 | .115 | -.19 | .021 | -.03 | .551 | -.13 | .069 | -.034 | .5 | -.133 | .065 |
|  | Migration Background |  |  |  |  |  |  |  |  |  |  |  |  |  |  |  |  |  |  |  |  |
|  | Ref.: No |  |  |  |  | 0 | . | . | . | 0 | . | . | . | 0 | . | . | . | 0 | . | . | . |
|  | 1. Generation |  |  |  |  | .027 | .395 | -.035 | .088 | .032 | .303 | -.029 | .094 | .102 | .001 | .044 | .16 | .103 | 0 | .045 | .16 |
|  | 2. Generation |  |  |  |  | .041 | .139 | -.013 | .096 | .037 | .187 | -.018 | .091 | .021 | .418 | -.03 | .072 | .019 | .457 | -.032 | .07 |
| Social Relationships | Children |  |  |  |  |  |  |  |  |  |  |  |  |  |  |  |  |  |  |  |  |
|  | Ref.: No |  |  |  |  |  |  |  |  | 0 | . | . | . | 0 | . | . | . | 0 | . | . | . |
|  | Yes |  |  |  |  |  |  |  |  | -.065 | .006 | -.112 | -.019 | -.018 | .436 | -.062 | .027 | -.005 | .82 | -.049 | .039 |
|  | Partnership Status |  |  |  |  |  |  |  |  |  |  |  |  |  |  |  |  |  |  |  |  |
|  | Ref.: Single |  |  |  |  |  |  |  |  | 0 | . | . | . | 0 | . | . | . | 0 | . | . | . |
|  | LAT |  |  |  |  |  |  |  |  | -.237 | 0 | -.297 | -.177 | -.142 | 0 | -.199 | -.085 | -.137 | 0 | -.194 | -.08 |
|  | Cohabitation |  |  |  |  |  |  |  |  | -.292 | 0 | -.34 | -.244 | -.165 | 0 | -.21 | -.119 | -.16 | 0 | -.205 | -.115 |
|  | Social Network |  |  |  |  |  |  |  |  |  |  |  |  |  |  |  |  |  |  |  |  |
|  | Ref.: No |  |  |  |  |  |  |  |  | 0 | . | . | . | 0 | . | . | . | 0 | . | . | . |
|  | Yes |  |  |  |  |  |  |  |  | -.074 | 0 | -.11 | -.037 | -.071 | 0 | -.105 | -.037 | -.066 | 0 | -.1 | -.031 |
| Health Status | Anxiety Disorder |  |  |  |  |  |  |  |  |  |  |  |  |  |  |  |  |  |  |  |  |
|  | Ref.: No |  |  |  |  |  |  |  |  |  |  |  |  | 0 | . | . | . | 0 | . | . | . |
|  | Yes |  |  |  |  |  |  |  |  |  |  |  |  | .153 | 0 | .098 | .207 | .113 | 0 | .058 | .167 |
|  | Depressive Mood |  |  |  |  |  |  |  |  |  |  |  |  |  |  |  |  |  |  |  |  |
|  | Ref.: No |  |  |  |  |  |  |  |  |  |  |  |  | 0 | . | . | . | 0 | . | . | . |
|  | Yes |  |  |  |  |  |  |  |  |  |  |  |  | .278 | 0 | .226 | .331 | .218 | 0 | .165 | .271 |
|  | Life Satisfaction |  |  |  |  |  |  |  |  |  |  |  |  | -.133 | 0 | -.143 | -.123 | -.116 | 0 | -.126 | -.105 |
|  | Covid19: Positive Aspects |  |  |  |  |  |  |  |  |  |  |  |  | -.019 | .019 | -.034 | -.003 | -.023 | .004 | -.039 | -.007 |
|  | Covid19: Personal Burden |  |  |  |  |  |  |  |  |  |  |  |  | .133 | 0 | .117 | .148 | .125 | 0 | .11 | .141 |
|  | Typ 2 Diabetes |  |  |  |  |  |  |  |  |  |  |  |  |  |  |  |  |  |  |  |  |
|  | Ref.: No |  |  |  |  |  |  |  |  |  |  |  |  | 0 | . | . | . | 0 | . | . | . |
|  | Yes |  |  |  |  |  |  |  |  |  |  |  |  | -.056 | .293 | -.161 | .048 | -.056 | .286 | -.16 | .047 |
|  | Hypertonie |  |  |  |  |  |  |  |  |  |  |  |  |  |  |  |  |  |  |  |  |
|  | Ref.: No |  |  |  |  |  |  |  |  |  |  |  |  | 0 | . | . | . | 0 | . | . | . |
|  | Yes |  |  |  |  |  |  |  |  |  |  |  |  | -.011 | .714 | -.067 | .046 | -.011 | .707 | -.067 | .045 |
|  | Cancer |  |  |  |  |  |  |  |  |  |  |  |  |  |  |  |  |  |  |  |  |
|  | Ref.: No |  |  |  |  |  |  |  |  |  |  |  |  | 0 | . | . | . | 0 | . | . | . |
|  | Yes |  |  |  |  |  |  |  |  |  |  |  |  | .021 | .728 | -.096 | .138 | .041 | .489 | -.075 | .157 |
|  | Asthma |  |  |  |  |  |  |  |  |  |  |  |  |  |  |  |  |  |  |  |  |
|  | Ref.: No |  |  |  |  |  |  |  |  |  |  |  |  | 0 | . | . | . | 0 | . | . | . |
|  | Yes |  |  |  |  |  |  |  |  |  |  |  |  | .048 | .096 | -.008 | .104 | .042 | .139 | -.014 | .098 |
|  | Obesity |  |  |  |  |  |  |  |  |  |  |  |  |  |  |  |  |  |  |  |  |
|  | Ref.: No |  |  |  |  |  |  |  |  |  |  |  |  | 0 | . | . | . | 0 | . | . | . |
|  | Yes |  |  |  |  |  |  |  |  |  |  |  |  | .001 | .954 | -.045 | .047 | -.018 | .435 | -.064 | .028 |
| Big Five | Neuroticism |  |  |  |  |  |  |  |  |  |  |  |  |  |  |  |  | .171 | 0 | .144 | .199 |
|  | Openness for Experience |  |  |  |  |  |  |  |  |  |  |  |  |  |  |  |  | .01 | .369 | -.011 | .031 |
|  | Agreeableness |  |  |  |  |  |  |  |  |  |  |  |  |  |  |  |  | -.045 | 0 | -.068 | -.022 |
|  | Conscientiousness |  |  |  |  |  |  |  |  |  |  |  |  |  |  |  |  | -.065 | 0 | -.086 | -.043 |
|  | Extraversion |  |  |  |  |  |  |  |  |  |  |  |  |  |  |  |  | -.022 | .069 | -.045 | .002 |
|  |  |  |  |  |  |  |  |  |  |  |  |  |  |  |  |  |  |  |  |  |  |
|  | Constant | 1.58 | 0 | 1.536 | 1.624 | 2.4 | 0 | 2.279 | 2.522 | 2.503 | 0 | 2.379 | 2.626 | 2.5 | 0 | 2.341 | 2.659 | 2.286 | 0 | 2.073 | 2.499 |
|  |  |  |  |  |  |  |  |  |  |  |  |  |  |  |  |  |  |  |  |  |  |
|  | Number of obs | 14,443 |  |  |  | 14,443 |  |  |  | 14,443 |  |  |  | 14,443 |  |  |  | 14,443 |  |  |  |
|  | R-squared | 0.016 |  |  |  | 0.058 |  |  |  | 0.071 |  |  |  | 0.179 |  |  |  | 0.192 |  |  |  |

*Note:* In model 1 we control for socio-demographic variables (education, satisfaction with household income, migrant background). In model 2 we also control for social relationships, namely partnership status, children and discuss personal matters with social network (with friends, acquaintances, neighbours or colleagues). In model 3, we also included health status, including dimensions of physical health, mental health and COVID 19 variables. The full model, model 4, also includes the Big Five personality variables.

*Source:* FReDA panel, waves 1 (autumn/winter 2021/22). Authors’ own calculations.

**Table A10 |** Stepwise Regression table (Social Loneliness)

|  |  | Base Model | | | | Model 1 | | | | Model 2 | | | | Model 3 | | | | Model 4 | | | |
| --- | --- | --- | --- | --- | --- | --- | --- | --- | --- | --- | --- | --- | --- | --- | --- | --- | --- | --- | --- | --- | --- |
|  |  | Coef. | p-value | 95 % CI | | Coef. | p-value | 95 % CI | | Coef. | p-value | 95 % CI | | Coef. | p-value | 95 % CI | | Coef. | p-value | 95 % CI | |
| Base Model | Age: |  |  |  |  |  |  |  |  |  |  |  |  |  |  |  |  |  |  |  |  |
|  | Ref.: 18-25 | 0 | . | . | . | 0 | . | . | . | 0 | . | . | . | 0 |  |  |  | 0 | . | . | . |
|  | 26-30 | -.061 | .087 | -.131 | .009 | .003 | .935 | -.067 | .073 | .06 | .096 | -.011 | .131 | .028 | .424 | -.041 | .097 | .035 | .323 | -.034 | .103 |
|  | 31-35 | .075 | .026 | .009 | .141 | .132 | 0 | .066 | .198 | .226 | 0 | .154 | .298 | .177 | 0 | .107 | .248 | .177 | 0 | .107 | .247 |
|  | 36-40 | .159 | 0 | .093 | .226 | .202 | 0 | .136 | .269 | .309 | 0 | .233 | .386 | .245 | 0 | .17 | .32 | .244 | 0 | .169 | .319 |
|  | 41-45 | .222 | 0 | .152 | .291 | .263 | 0 | .194 | .333 | .371 | 0 | .292 | .451 | .285 | 0 | .206 | .364 | .293 | 0 | .214 | .372 |
|  | 46-51 | .258 | 0 | .186 | .33 | .289 | 0 | .218 | .36 | .386 | 0 | .304 | .468 | .295 | 0 | .213 | .377 | .32 | 0 | .238 | .402 |
|  | Sex |  |  |  |  |  |  |  |  |  |  |  |  |  |  |  |  |  |  |  |  |
|  | Ref.: Male | 0 | . | . | . | 0 | . | . | . | 0 | . | . | . | 0 |  |  |  | 0 | . | . | . |
|  | Female | -.077 | 0 | -.118 | -.036 | -.097 | 0 | -.138 | -.057 | -.032 | .123 | -.072 | .009 | -.049 | .016 | -.089 | -.009 | -.004 | .855 | -.044 | .036 |
| Socio-Demographic Variables | Satisfaction with HH-Income |  |  |  |  | -.165 | 0 | -.182 | -.148 | -.154 | 0 | -.171 | -.137 | -.076 | 0 | -.094 | -.058 | -.071 | 0 | -.089 | -.054 |
|  | Educational Background |  |  |  |  |  |  |  |  |  |  |  |  |  |  |  |  |  |  |  |  |
|  | Ref.: low |  |  |  |  | 0 | . | . | . | 0 | . | . | . | 0 | . | . | . | 0 | . | . | . |
|  | middle |  |  |  |  | -.15 | .016 | -.272 | -.027 | -.087 | .16 | -.207 | .034 | -.041 | .491 | -.159 | .076 | -.044 | .454 | -.161 | .072 |
|  | high |  |  |  |  | -.311 | 0 | -.434 | -.188 | -.211 | .001 | -.333 | -.089 | -.145 | .016 | -.264 | -.027 | -.124 | .039 | -.242 | -.006 |
|  | Migration Background |  |  |  |  |  |  |  |  |  |  |  |  |  |  |  |  |  |  |  |  |
|  | Ref.: No |  |  |  |  | 0 | . | . | . | 0 | . | . | . | 0 | . | . | . | 0 | . | . | . |
|  | 1. Generation |  |  |  |  | .236 | 0 | .164 | .308 | .224 | 0 | .153 | .295 | .295 | 0 | .226 | .364 | .311 | 0 | .242 | .379 |
|  | 2. Generation |  |  |  |  | .08 | .014 | .016 | .144 | .081 | .012 | .018 | .144 | .07 | .025 | .009 | .131 | .075 | .015 | .014 | .136 |
| Social Relationships | Children |  |  |  |  |  |  |  |  |  |  |  |  |  |  |  |  |  |  |  |  |
|  | Ref.: No |  |  |  |  |  |  |  |  | 0 | . | . | . | 0 | . | . | . | 0 | . | . | . |
|  | Yes |  |  |  |  |  |  |  |  | -.053 | .054 | -.107 | .001 | .006 | .812 | -.046 | .059 | .018 | .5 | -.034 | .07 |
|  | Partnership Status |  |  |  |  |  |  |  |  |  |  |  |  |  |  |  |  |  |  |  |  |
|  | Ref.: Single |  |  |  |  |  |  |  |  | 0 | . | . | . | 0 | . | . | . | 0 | . | . | . |
|  | LAT |  |  |  |  |  |  |  |  | -.349 | 0 | -.419 | -.28 | -.253 | 0 | -.321 | -.185 | -.227 | 0 | -.294 | -.16 |
|  | Cohabitation |  |  |  |  |  |  |  |  | -.34 | 0 | -.395 | -.285 | -.216 | 0 | -.27 | -.161 | -.208 | 0 | -.262 | -.154 |
|  | Social Network |  |  |  |  |  |  |  |  |  |  |  |  |  |  |  |  |  |  |  |  |
|  | Ref.: No |  |  |  |  |  |  |  |  | 0 | . | . | . | 0 | . | . | . | 0 | . | . | . |
|  | Yes |  |  |  |  |  |  |  |  | -.375 | 0 | -.417 | -.333 | -.362 | 0 | -.402 | -.321 | -.324 | 0 | -.365 | -.283 |
| Health Status | Anxiety Disorder |  |  |  |  |  |  |  |  |  |  |  |  |  |  |  |  |  |  |  |  |
|  | Ref.: No |  |  |  |  |  |  |  |  |  |  |  |  | 0 | . | . | . | 0 | . | . | . |
|  | Yes |  |  |  |  |  |  |  |  |  |  |  |  | .116 | .001 | .051 | .182 | .102 | .002 | .037 | .168 |
|  | Depressive Mood |  |  |  |  |  |  |  |  |  |  |  |  |  |  |  |  |  |  |  |  |
|  | Ref.: No |  |  |  |  |  |  |  |  |  |  |  |  | 0 | . | . | . | 0 | . | . | . |
|  | Yes |  |  |  |  |  |  |  |  |  |  |  |  | .199 | 0 | .136 | .262 | .16 | 0 | .097 | .223 |
|  | Life Satisfaction |  |  |  |  |  |  |  |  |  |  |  |  | -.141 | 0 | -.153 | -.129 | -.118 | 0 | -.131 | -.106 |
|  | Covid19: Positive Aspects |  |  |  |  |  |  |  |  |  |  |  |  | -.04 | 0 | -.059 | -.022 | -.035 | 0 | -.054 | -.017 |
|  | Covid19: Personal Burden |  |  |  |  |  |  |  |  |  |  |  |  | .006 | .495 | -.012 | .025 | .012 | .184 | -.006 | .031 |
|  | Typ 2 Diabetes |  |  |  |  |  |  |  |  |  |  |  |  |  |  |  |  |  |  |  |  |
|  | Ref.: No |  |  |  |  |  |  |  |  |  |  |  |  | 0 | . | . | . | 0 | . | . | . |
|  | Yes |  |  |  |  |  |  |  |  |  |  |  |  | -.056 | .378 | -.181 | .069 | -.051 | .416 | -.175 | .072 |
|  | Hypertonie |  |  |  |  |  |  |  |  |  |  |  |  |  |  |  |  |  |  |  |  |
|  | Ref.: No |  |  |  |  |  |  |  |  |  |  |  |  | 0 | . | . | . | 0 | . | . | . |
|  | Yes |  |  |  |  |  |  |  |  |  |  |  |  | .048 | .16 | -.019 | .116 | .043 | .21 | -.024 | .11 |
|  | Cancer |  |  |  |  |  |  |  |  |  |  |  |  |  |  |  |  |  |  |  |  |
|  | Ref.: No |  |  |  |  |  |  |  |  |  |  |  |  | 0 | . | . | . | 0 | . | . | . |
|  | Yes |  |  |  |  |  |  |  |  |  |  |  |  | .006 | .934 | -.134 | .145 | .021 | .764 | -.117 | .159 |
|  | Asthma |  |  |  |  |  |  |  |  |  |  |  |  |  |  |  |  |  |  |  |  |
|  | Ref.: No |  |  |  |  |  |  |  |  |  |  |  |  | 0 | . | . | . | 0 | . | . | . |
|  | Yes |  |  |  |  |  |  |  |  |  |  |  |  | .044 | .193 | -.022 | .111 | .057 | .092 | -.009 | .123 |
|  | Obesity |  |  |  |  |  |  |  |  |  |  |  |  |  |  |  |  |  |  |  |  |
|  | Ref.: No |  |  |  |  |  |  |  |  |  |  |  |  | 0 | . | . | . | 0 | . | . | . |
|  | Yes |  |  |  |  |  |  |  |  |  |  |  |  | .045 | .108 | -.01 | .1 | .024 | .398 | -.031 | .078 |
| Big Five | Neuroticism |  |  |  |  |  |  |  |  |  |  |  |  |  |  |  |  | .071 | 0 | .038 | .104 |
|  | Openness for Experience |  |  |  |  |  |  |  |  |  |  |  |  |  |  |  |  | -.043 | .001 | -.068 | -.018 |
|  | Agreeableness |  |  |  |  |  |  |  |  |  |  |  |  |  |  |  |  | -.193 | 0 | -.22 | -.166 |
|  | Conscientiousness |  |  |  |  |  |  |  |  |  |  |  |  |  |  |  |  | -.042 | .002 | -.068 | -.016 |
|  | Extraversion |  |  |  |  |  |  |  |  |  |  |  |  |  |  |  |  | -.082 | 0 | -.11 | -.054 |
|  |  |  |  |  |  |  |  |  |  |  |  |  |  |  |  |  |  |  |  |  |  |
|  | Constant | 1.388 | 0 | 1.336 | 1.44 | 2.32 | 0 | 2.179 | 2.462 | 2.598 | 0 | 2.456 | 2.741 | 3.206 | 0 | 3.016 | 3.396 | 3.958 | 0 | 3.704 | 4.212 |
|  |  |  |  |  |  |  |  |  |  |  |  |  |  |  |  |  |  |  |  |  |  |
|  | Number of obs | 14,443 |  |  |  | 14,443 |  |  |  | 14,443 |  |  |  | 14,443 |  |  |  | 14,443 |  |  |  |
|  | R-squared | 0.009 |  |  |  | 0.048 |  |  |  | 0.077 |  |  |  | 0.131 |  |  |  | 0.149 |  |  |  |

Note: In model 1 we control for socio-demographic variables (education, satisfaction with household income, migrant background). In model 2 we also control for social relationships, namely partnership status, children and discuss personal matters with social network (with friends, acquaintances, neighbours or colleagues). In model 3, we also included health status, including dimensions of physical health, mental health and COVID 19 variables. The full model, model 4, also includes the Big Five personality variables.

Source: FReDA panel, waves 1 (autumn/winter 2021/22). Authors’ own calculations.

**Table A11 |** Regression Tables. Full Model for Overall Loneliness, Emotional Loneliness and Social Loneliness, stratified by age groups.

| *Overall Loneliness* |  |  |  | |
| --- | --- | --- | --- | --- |
| Age | Predicted Value | p-value | 95% Confidence Interval | |
| 18-25 | 1.39 | 0.000 | 1.353 | 1.433 |
| 26-30 | 1.394 | 0.000 | 1.355 | 1.434 |
| 31-35 | 1.454 | 0.000 | 1.419 | 1.488 |
| 36-40 | 1.454 | 0.000 | 1.418 | 1.490 |
| 41-45 | 1.467 | 0.000 | 1.429 | 1.508 |
| 46-52 | 1.483 | 0.000 | 1.441 | 1.526 |
| *Emotional Loneliness* |  |  |  | |
| Age | Predicted Value | p-value | 95% Confidence Interval | |
| 18-25 | 1.509 | 0.000 | 1.465 | 1.552 |
| 26-30 | 1.477 | 0.000 | 1.434 | 1.520 |
| 31-35 | 1.453 | 0.000 | 1.416 | 1.490 |
| 36-40 | 1.387 | 0.000 | 1.348 | 1.426 |
| 41-45 | 1.367 | 0.000 | 1.324 | 1.410 |
| 46-52 | 1.370 | 0.000 | 1.323 | 1.415 |
| *Social Loneliness* |  |  |  | |
| Age | Predicted Value | p-value | 95% Confidence Interval | |
| 18-25 | 1.277 | 0.000 | 1.225 | 1.329 |
| 26-30 | 1.312 | 0.000 | 1.261 | 1.363 |
| 31-35 | 1.454 | 0.000 | 1.410 | 1.499 |
| 36-40 | 1.522 | 0.000 | 1.475 | 1.568 |
| 41-45 | 1.570 | 0.000 | 1.520 | 1.621 |
| 46-52 | 1.597 | 0.000 | 1.543 | 1.652 |

*Note:* Estimated predicted values are reported at the mean values of all covariates, meaning that the coefficients represent the expected outcome when all independent variables are set to their sample means within each age group.

*Source:* FReDA Panel, wave 1 (autumn/winter 2021/22). Authors’ own calculations.

**Table A12 |** Regression table (z-standardized), overall loneliness

|  | Coef. | p-value | [95% Conf | Interval] |
| --- | --- | --- | --- | --- |
| Age : |  |  |  |  |
| Ref.: 18-25 | 0 | . | . | . |
| 26-30 | .001 | .963 | -.052 | .054 |
| 31-35 | .06 | .029 | .006 | .115 |
| 36-40 | .061 | .038 | .003 | .119 |
| 41-45 | .075 | .015 | .015 | .136 |
| 46-51 | .09 | .005 | .027 | .153 |
| Gender |  |  |  |  |
| Ref.: Male | 0 | . | . | . |
| Female | .041 | .009 | .01 | .072 |
| Satisfaction with HH-Income | -.064 | 0 | -.078 | -.051 |
| Educational Background |  |  |  |  |
| Ref.: low | 0 | . | . | . |
| middle | .002 | .96 | -.088 | .092 |
| high | -.079 | .089 | -.17 | .012 |
| Migration Background |  |  |  |  |
| Ref.: No | 0 | . | . | . |
| 1. Generation | .207 | 0 | .154 | .26 |
| 2. Generation | .047 | .049 | 0 | .094 |
| Children |  |  |  |  |
| Ref.: No | 0 | . | . | . |
| Yes | .006 | .755 | -.034 | .047 |
| Partnership Status |  |  |  |  |
| Ref.: Single | 0 | . | . | . |
| LAT | -.182 | 0 | -.234 | -.13 |
| Cohabitation | -.184 | 0 | -.226 | -.142 |
| Social Network |  |  |  |  |
| Ref.: No | 0 | . | . | . |
| Yes | -.195 | 0 | -.226 | -.163 |
| Anxiety |  |  |  |  |
| Ref.: No | 0 | . | . | . |
| Yes | .107 | 0 | .057 | .158 |
| Depressive Mood |  |  |  |  |
| Ref.: No | 0 | . | . | . |
| Yes | .189 | 0 | .14 | .238 |
| Life Satisfaction | -.117 | 0 | -.127 | -.107 |
| Covid19: Positive Aspects | -.029 | 0 | -.043 | -.015 |
| Covid19: Personal Burden | .069 | 0 | .055 | .083 |
| Typ 2 Diabetes |  |  |  |  |
| Ref.: No | 0 | . | . | . |
| Yes | -.054 | .27 | -.149 | .042 |
| Hypertonie |  |  |  |  |
| Ref.: No | 0 | . | . | . |
| Yes | .016 | .545 | -.036 | .068 |
| Cancer |  |  |  |  |
| Ref.: No | 0 | . | . | . |
| Yes | .031 | .569 | -.076 | .138 |
| Asthma |  |  |  |  |
| Ref.: No | 0 | . | . | . |
| Yes | .049 | .059 | -.002 | .101 |
| Obesity |  |  |  |  |
| Ref.: No | 0 | . | . | . |
| Yes | .003 | .903 | -.04 | .045 |
| Neuroticism | .121 | 0 | .095 | .146 |
| Openness for Experience | -.017 | .095 | -.036 | .003 |
| Agreeableness | -.119 | 0 | -.14 | -.098 |
| Conscientiousness | -.053 | 0 | -.073 | -.033 |
| Extraversion | -.052 | 0 | -.073 | -.03 |
| Constant | 1.681 | 0 | 1.484 | 1.877 |
| N | 14,443 | | | |
| R squared | 0.197 | | | |

**Table A13 |** Regression table (z-standardized), emotional loneliness

|  | Coef. | p-value | [95% Conf | Interval] |
| --- | --- | --- | --- | --- |
| Age : |  |  |  |  |
| Ref.: 18-25 | 0 | . | . | . |
| 26-30 | -.03 | .275 | -.083 | .024 |
| 31-35 | -.052 | .064 | -.106 | .003 |
| 36-40 | -.113 | 0 | -.171 | -.055 |
| 41-45 | -.131 | 0 | -.192 | -.07 |
| 46-51 | -.129 | 0 | -.192 | -.065 |
| Gender |  |  |  |  |
| Ref.: Male | 0 | . | . | . |
| Female | .08 | 0 | .048 | .111 |
| Satisfaction with HH-Income | -.053 | 0 | -.067 | -.04 |
| Educational Background |  |  |  |  |
| Ref.: low | 0 | . | . | . |
| middle | .045 | .326 | -.045 | .136 |
| high | -.031 | .5 | -.123 | .06 |
| Migration Background |  |  |  |  |
| Ref.: No | 0 | . | . | . |
| 1. Generation | .095 | 0 | .042 | .148 |
| 2. Generation | .018 | .457 | -.029 | .065 |
| Children |  |  |  |  |
| Ref.: No | 0 | . | . | . |
| Yes | -.005 | .82 | -.045 | .036 |
| Partnership Status |  |  |  |  |
| Ref.: Single | 0 | . | . | . |
| LAT | -.127 | 0 | -.179 | -.074 |
| Cohabitation | -.148 | 0 | -.19 | -.106 |
| Social Network |  |  |  |  |
| Ref.: No | 0 | . | . | . |
| Yes | -.061 | 0 | -.092 | -.029 |
| Anxiety |  |  |  |  |
| Ref.: No | 0 | . | . | . |
| Yes | .104 | 0 | .053 | .155 |
| Depressive Mood |  |  |  |  |
| Ref.: No | 0 | . | . | . |
| Yes | .202 | 0 | .153 | .251 |
| Life Satisfaction | -.107 | 0 | -.117 | -.098 |
| Covid19: Positive Aspects | -.021 | .004 | -.036 | -.007 |
| Covid19: Personal Burden | .116 | 0 | .102 | .13 |
| Typ 2 Diabetes |  |  |  |  |
| Ref.: No | 0 | . | . | . |
| Yes | -.052 | .286 | -.148 | .044 |
| Hypertonie |  |  |  |  |
| Ref.: No | 0 | . | . | . |
| Yes | -.01 | .707 | -.062 | .042 |
| Cancer |  |  |  |  |
| Ref.: No | 0 | . | . | . |
| Yes | .038 | .489 | -.069 | .145 |
| Asthma |  |  |  |  |
| Ref.: No | 0 | . | . | . |
| Yes | .039 | .139 | -.013 | .09 |
| Obesity |  |  |  |  |
| Ref.: No | 0 | . | . | . |
| Yes | -.017 | .435 | -.059 | .026 |
| Neuroticism | .158 | 0 | .133 | .184 |
| Openness for Experience | .009 | .369 | -.011 | .028 |
| Agreeableness | -.041 | 0 | -.063 | -.02 |
| Conscientiousness | -.06 | 0 | -.08 | -.04 |
| Extraversion | -.02 | .069 | -.042 | .002 |
| Constant | .79 | 0 | .593 | .988 |
| N | 14,443 | | | |
| R sqaured | 0.192 | | | |

**Table A14 |** Regression table (z-standardized), social loneliness

|  | Coef. | p-value | [95% Conf | Interval] |
| --- | --- | --- | --- | --- |
| Age : |  |  |  |  |
| Ref.: 18-25 | 0 | . | . | . |
| 26-30 | .028 | .323 | -.027 | .082 |
| 31-35 | .141 | 0 | .085 | .197 |
| 36-40 | .195 | 0 | .135 | .254 |
| 41-45 | .234 | 0 | .171 | .296 |
| 46-51 | .255 | 0 | .19 | .32 |
| Gender |  |  |  |  |
| Ref.: Male | 0 | . | . | . |
| Female | -.003 | .855 | -.035 | .029 |
| Satisfaction with HH-Income | -.057 | 0 | -.071 | -.043 |
| Educational Background |  |  |  |  |
| Ref.: low | 0 | . | . | . |
| middle | -.035 | .454 | -.128 | .057 |
| high | -.099 | .039 | -.193 | -.005 |
| Migration Background |  |  |  |  |
| Ref.: No | 0 | . | . | . |
| 1. Generation | .248 | 0 | .193 | .302 |
| 2. Generation | .06 | .015 | .012 | .108 |
| Children |  |  |  |  |
| Ref.: No | 0 | . | . | . |
| Yes | .014 | .5 | -.027 | .056 |
| Partnership Status |  |  |  |  |
| Ref.: Single | 0 | . | . | . |
| LAT | -.181 | 0 | -.234 | -.127 |
| Cohabitation | -.166 | 0 | -.209 | -.123 |
| Social Network |  |  |  |  |
| Ref.: No | 0 | . | . | . |
| Yes | -.258 | 0 | -.291 | -.226 |
| Anxiety |  |  |  |  |
| Ref.: No | 0 | . | . | . |
| Yes | .082 | .002 | .03 | .134 |
| Depressive Mood |  |  |  |  |
| Ref.: No | 0 | . | . | . |
| Yes | .127 | 0 | .077 | .178 |
| Life Satisfaction | -.094 | 0 | -.104 | -.084 |
| Covid19: Positive Aspects | -.028 | 0 | -.043 | -.013 |
| Covid19: Personal Burden | .01 | .184 | -.005 | .024 |
| Typ 2 Diabetes |  |  |  |  |
| Ref.: No | 0 | . | . | . |
| Yes | -.041 | .416 | -.139 | .058 |
| Hyptertonie |  |  |  |  |
| Ref.: No | 0 | . | . | . |
| Yes | .034 | .21 | -.019 | .087 |
| Cancer |  |  |  |  |
| Ref.: No | 0 | . | . | . |
| Yes | .017 | .764 | -.093 | .127 |
| Asthma |  |  |  |  |
| Ref.: No | 0 | . | . | . |
| Yes | .045 | .092 | -.007 | .098 |
| Obesity |  |  |  |  |
| Ref.: No | 0 | . | . | . |
| Yes | .019 | .398 | -.025 | .062 |
| Neuroticism | .056 | 0 | .03 | .083 |
| Openness for Experience | -.034 | .001 | -.054 | -.014 |
| Agreeableness | -.154 | 0 | -.176 | -.132 |
| Conscientiousness | -.033 | .002 | -.054 | -.013 |
| Extraversion | -.065 | 0 | -.088 | -.043 |
| Constant | 2.001 | 0 | 1.798 | 2.203 |
| N | 14,443 | | | |
| R squared | 0.149 | | | |

**Table A15 |** Regression Tables. Full Model for Overall Loneliness, Emotional Loneliness and Social Loneliness, stratified by age groups and gender.

| *Overall Loneliness* | | | | | | | | | | | | | | | |
| --- | --- | --- | --- | --- | --- | --- | --- | --- | --- | --- | --- | --- | --- | --- | --- |
| Age | | |  | | | Predicted Value | | | p-value | | | 95% Confidence Interval | | | |
| 18-25 | | | Male | | | 1.370 | | | 0.000 | | | 1.326 | | | 1.415 |
|  |  |  | Female | | | 1.411 | | | 0.000 | | | 1.369 | | | 1.453 |
| 26-30 | | | Male | | | 1.371 | | | 0.000 | | | 1.328 | | | 1.415 |
|  |  |  | Female | | | 1.413 | | | 0.000 | | | 1.371 | | | 1.454 |
| 31-35 | | | Male | | | 1.431 | | | 0.000 | | | 1.392 | | | 1.469 |
|  |  |  | Female | | | 1.472 | | | 0.000 | | | 1.435 | | | 1.509 |
| 36-40 | | | Male | | | 1.431 | | | 0.000 | | | 1.392 | | | 1.471 |
|  |  |  | Female | | | 1.473 | | | 0.000 | | | 1.434 | | | 1.511 |
| 41-45 | | | Male | | | 1.446 | | | 0.000 | | | 1.403 | | | 1.489 |
|  |  |  | Female | | | 1.487 | | | 0.000 | | | 1.445 | | | 1.529 |
| 46-52 | | | Male | | | 1.461 | | | 0.000 | | | 1.415 | | | 1.506 |
|  |  |  | Female | | | 1.502 | | | 0.000 | | | 1.457 | | | 1.547 |
| *Emotional Loneliness* | | | | | | | | | | | | | | | |
| Age | |  | | | Predicted Value | | | p-value | | | 95% Confidence Interval | | | | |
| 18-25 | | Male | | | 1.461 | | | 0.000 | | | 1.413 | | | 1.509 | |
|  |  | Female | | | 1.547 | | | 0.000 | | | 1.502 | | | 1.593 | |
| 26-30 | | Male | | | 1.429 | | | 0.000 | | | 1.381 | | | 1.476 | |
|  |  | Female | | | 1.515 | | | 0.000 | | | 1.470 | | | 1.560 | |
| 31-35 | | Male | | | 1.405 | | | 0.000 | | | 1.364 | | | 1.447 | |
|  |  | Female | | | 1.491 | | | 0.000 | | | 1.451 | | | 1.532 | |
| 36-40 | | Male | | | 1.339 | | | 0.000 | | | 1.296 | | | 1.382 | |
|  |  | Female | | | 1.425 | | | 0.000 | | | 1.383 | | | 1.467 | |
| 41-45 | | Male | | | 1.319 | | | 0.000 | | | 1.273 | | | 1.365 | |
|  |  | Female | | | 1.405 | | | 0.000 | | | 1.359 | | | 1.451 | |
| 46-52 | | Male | | | 1.322 | | | 0.000 | | | 1.272 | | | 1.371 | |
|  |  | Female | | | 1.408 | | | 0.000 | | | 1.359 | | | 1.457 | |
| *Social Loneliness* | | | | | | | | | | | | | | | |
| Age |  | | | Predicted Value | | | p-value | | | 95% Confidence Interval | | | | | |
| 18-25 | Male | | | 1.279 | | | 0.000 | | | 1.222 | | | 1.337 | | |
|  | Female | | | 1.276 | | | 0.000 | | | 1.221 | | | 1.330 | | |
| 26-30 | Male | | | 1.314 | | | 0.000 | | | 1.258 | | | 1.371 | | |
|  | Female | | | 1.310 | | | 0.000 | | | 1.256 | | | 1.364 | | |
| 31-35 | Male | | | 1.456 | | | 0.000 | | | 1.407 | | | 1.506 | | |
|  | Female | | | 1.452 | | | 0.000 | | | 1.404 | | | 1.500 | | |
| 36-40 | Male | | | 1.524 | | | 0.000 | | | 1.472 | | | 1.575 | | |
|  | Female | | | 1.520 | | | 0.000 | | | 1.470 | | | 1.570 | | |
| 41-45 | Male | | | 1.572 | | | 0.000 | | | 1.517 | | | 1.628 | | |
|  | Female | | | 1.569 | | | 0.000 | | | 1.514 | | | 1.623 | | |
| 46-52 | Male | | | 1.600 | | | 0.000 | | | 1.541 | | | 1.658 | | |
|  | Female | | | 1.596 | | | 0.000 | | | 1.538 | | | 1.654 | | |

*Note:* Estimated predicted values are reported at the mean values of all covariates, meaning that the coefficients represent the expected outcome when all independent variables are set to their sample means within each age group, separately for each gender.

*Source:* FReDA Panel, wave 1 (autumn/winter 2021/22). Authors’ own calculations.

**Table A16 |** Regression Table. Full Model by Gender. Emotional Loneliness.

|  | Male | | | | Female | | | |
| --- | --- | --- | --- | --- | --- | --- | --- | --- |
|  | Coef. | p-value | [95% Conf | Interval] | Coef. | p-value | [95% Conf | Interval] |
| Age: |  |  |  |  |  |  |  |  |
| Ref.: 18-25 | 0 | . | . | . | 0 | . | . | . |
| 26-30 | -.03 | .488 | -.116 | .056 | -.036 | .369 | -.114 | .042 |
| 31-35 | -.084 | .054 | -.17 | .001 | -.036 | .39 | -.118 | .046 |
| 36-40 | -.082 | .079 | -.174 | .009 | -.156 | 0 | -.243 | -.07 |
| 41-45 | -.147 | .003 | -.244 | -.05 | -.142 | .002 | -.233 | -.051 |
| 46-51 | -.109 | .036 | -.21 | -.007 | -.168 | 0 | -.262 | -.074 |
| Satisfaction with HH-Income | -.051 | 0 | -.073 | -.029 | -.063 | 0 | -.082 | -.043 |
| Educational Background |  |  |  |  |  |  |  |  |
| Ref.: low | 0 | . | . | . | 0 | . | . | . |
| middle | -.025 | .73 | -.115 | .164 | .076 | .272 | -.06 | .213 |
| high | -.075 | .229 | -.217 | .067 | .009 | .897 | -.129 | .147 |
| Migration Background |  |  |  |  |  |  |  |  |
| Ref.: No | 0 | . | . | . | 0 | . | . | . |
| 1. Generation | .106 | .017 | .019 | .193 | .1 | .011 | .023 | .177 |
| 2. Generation | .007 | .86 | -.068 | .081 | .027 | .454 | -.043 | .097 |
| Children |  |  |  |  |  |  |  |  |
| Ref.: No | 0 | . | . | . | 0 | . | . | . |
| Yes | -.025 | .452 | -.09 | .04 | .012 | .684 | -.048 | .073 |
| Partnership Status |  |  |  |  |  |  |  |  |
| Ref.: Single | 0 | . | . | . | 0 | . | . | . |
| LAT | -.122 | .005 | -.207 | -.038 | -.135 | .001 | -.212 | -.057 |
| Cohabitation | -.152 | 0 | -.22 | -.085 | -.159 | 0 | -.221 | -.097 |
| Social Network |  |  |  |  |  |  |  |  |
| Ref.: No | 0 | . | . | . | 0 | . | . | . |
| Yes | -.036 | .146 | -.085 | .013 | -.092 | 0 | -.14 | -.044 |
| Anxiety |  |  |  |  |  |  |  |  |
| Ref.: No | 0 | . | . | . | 0 | . | . | . |
| Yes | .123 | .01 | .03 | .217 | .11 | .002 | .042 | .179 |
| Depressive Mood |  |  |  |  |  |  |  |  |
| Ref.: No | 0 | . | . | . | 0 | . | . | . |
| Yes | .241 | 0 | .156 | .327 | .21 | 0 | .141 | .278 |
| Life Satisfaction | -.123 | 0 | -.138 | -.107 | -.11 | 0 | -.124 | -.096 |
| Covid19: Positive Aspects | -.03 | .008 | -.052 | -.008 | -.017 | .128 | -.039 | .005 |
| Covid19: Personal Burden | .136 | 0 | .114 | .158 | .116 | 0 | .094 | .137 |
| Typ 2 Diabetes |  |  |  |  |  |  |  |  |
| Ref.: No | 0 | . | . | . | 0 | . | . | . |
| Yes | -.026 | .758 | -.194 | .141 | -.081 | .235 | -.214 | .052 |
| Hypertonie |  |  |  |  |  |  |  |  |
| Ref.: No | 0 | . | . | . | 0 | . | . | . |
| Yes | -.035 | .355 | -.11 | .039 | .022 | .622 | -.064 | .107 |
| Cancer |  |  |  |  |  |  |  |  |
| Ref.: No | 0 | . | . | . | 0 | . | . | . |
| Yes | .067 | .508 | -.132 | .267 | .029 | .693 | -.115 | .173 |
| Asthma |  |  |  |  |  |  |  |  |
| Ref.: No | 0 | . | . | . | 0 | . | . | . |
| Yes | .003 | .935 | -.078 | .085 | .07 | .072 | -.006 | .146 |
| Obesity |  |  |  |  |  |  |  |  |
| Ref.: No | 0 | . | . | . | 0 | . | . | . |
| Yes | -.048 | .16 | -.115 | .019 | .003 | .918 | -.06 | .067 |
| Neuroticism | .181 | 0 | .141 | .22 | .162 | 0 | .123 | .201 |
| Openness for Experience | -.005 | .738 | -.036 | .025 | .022 | .141 | -.007 | .051 |
| Agreeableness | -.037 | .026 | -.071 | -.004 | -.054 | .001 | -.086 | -.022 |
| Conscientiousness | -.069 | 0 | -.101 | -.037 | -.06 | .0 | -.09 | -.03 |
| Extraversion | -.025 | .16 | -.06 | .01 | -.02 | .204 | -.052 | .011 |
| Constant | 2.342 | 0 | 2.036 | 2.648 | 2.338 | 0 | 2.034 | 2.643 |
|  |  |  |  |  |  |  |  |  |
| N | 6,433 | | | | 8,010 | | | |
| R squared | 0.214 | | | | 0.172 | | | |

*Source:* FReDA Panel, wave 1 (autumn/winter 2021/22). Authors’ own calculations.

**Table A17 |** Regression Table. Full Model by Gender. Social Loneliness.

|  | Male | | | | Female | | | |
| --- | --- | --- | --- | --- | --- | --- | --- | --- |
|  | Coef. | p-value | [95% Conf | Interval] | Coef. | p-value | [95% Conf | Interval] |
| Age: |  |  |  |  |  |  |  |  |
| Ref.: 18-25 | 0 | . | . | . | 0 | . | . | . |
| 26-30 | .056 | .28 | -.046 | .158 | .017 | .72 | -.076 | .11 |
| 31-35 | .22 | 0 | .119 | .322 | .141 | .005 | .043 | .239 |
| 36-40 | .271 | 0 | .162 | .379 | .223 | 0 | .119 | .326 |
| 41-45 | .346 | 0 | .231 | .461 | .248 | 0 | .139 | .356 |
| 46-51 | .393 | 0 | .273 | .513 | .267 | 0 | .155 | .379 |
| Satisfaction with HH-Income | -.066 | 0 | -.093 | -.04 | -.076 | 0 | -.099 | -.052 |
| Educational Background |  |  |  |  |  |  |  |  |
| Ref.: low | 0 | . | . | . | 0 | . | . | . |
| middle | -.145 | .085 | -.311 | .02 | .05 | .545 | -.113 | .213 |
| high | -.23 | .007 | -.397 | -.062 | -.026 | .756 | -.191 | .139 |
| Migration Background |  |  |  |  |  |  |  |  |
| Ref.: No | 0 | . | . | . | 0 | . | . | . |
| 1. Generation | .265 | 0 | .162 | .368 | .344 | 0 | .252 | .436 |
| 2. Generation | .041 | .361 | -.047 | .129 | .101 | .018 | .017 | .184 |
| Children |  |  |  |  |  |  |  |  |
| Ref.: No | 0 | . | . | . | 0 | . | . | . |
| Yes | -.004 | .922 | -.081 | .073 | .04 | .277 | -.032 | .112 |
| Partnership Status |  |  |  |  |  |  |  |  |
| Ref.: Single | 0 | . | . | . | 0 | . | . | . |
| LAT | -.251 | 0 | -.351 | -.151 | -.192 | 0 | -.285 | -.1 |
| Cohabitation | -.274 | 0 | -.353 | -.194 | -.15 | 0 | -.225 | -.076 |
| Social Network | 0 | . | . | . | 0 | . | . | . |
| Ref.: No |  |  |  |  |  |  |  |  |
| Yes | -.344 | 0 | -.402 | -.286 | -.307 | 0 | -.365 | -.25 |
| Anxiety |  |  |  |  |  |  |  |  |
| Ref.: No | 0 | . | . | . | 0 | . | . | . |
| Yes | .087 | .121 | -.023 | .198 | .109 | .009 | .028 | .191 |
| Depressive Mood |  |  |  |  |  |  |  |  |
| Ref.: No | 0 | . | . | . | 0 | . | . | . |
| Yes | .175 | .001 | .074 | .276 | .154 | 0 | .072 | .236 |
| Life Satisfaction | -.12 | 0 | -.138 | -.102 | -.118 | 0 | -.135 | -.101 |
| Covid19: Positive Aspects | -.018 | .189 | -.044 | .009 | -.048 | 0 | -.074 | -.022 |
| Covid19: Personal Burden | .018 | .174 | -.008 | .044 | .009 | .509 | -.017 | .034 |
| Typ 2 Diabetes |  |  |  |  |  |  |  |  |
| Ref.: No | 0 | . | . | . | 0 | . | . | . |
| Yes | -.028 | .781 | -.226 | .17 | -.074 | .366 | -.233 | .086 |
| Hypertonie |  |  |  |  |  |  |  |  |
| Ref.: No | 0 | . | . | . | 0 | . | . | . |
| Yes | .067 | .134 | -.021 | .155 | .02 | .706 | -.083 | .122 |
| Cancer |  |  |  |  |  |  |  |  |
| Ref.: No | 0 | . | . | . | 0 | . | . | . |
| Yes | -.086 | .475 | -.322 | .15 | .079 | .368 | -.093 | .251 |
| Asthma |  |  |  |  |  |  |  |  |
| Ref.: No | 0 | . | . | . | 0 | . | . | . |
| Yes | -.008 | .878 | -.104 | .089 | .111 | .017 | .02 | .201 |
| Obesity |  |  |  |  |  |  |  |  |
| Ref.: No | 0 | . | . | . | 0 | . | . | . |
| Yes | .008 | .839 | -.071 | .087 | .031 | .417 | -.044 | .107 |
| Neuroticism | .085 | 0 | .038 | .131 | .058 | .015 | .011 | .104 |
| Openness for Experience | -.086 | 0 | -.122 | -.05 | -.008 | .638 | -.043 | .026 |
| Agreeableness | -.217 | 0 | -.256 | -.178 | -.175 | 0 | -.213 | -.137 |
| Conscientiousness | -.031 | .11 | -.069 | .007 | -.05 | .006 | -.086 | -.015 |
| Extraversion | -.078 | 0 | -.119 | -.037 | -.086 | 0 | -.124 | -.048 |
| Constant | 4.149 | 0 | 3.787 | 4.511 | 3.783 | 0 | 3.419 | 4.147 |
|  |  |  |  |  |  |  |  |  |
| N | 6,433 | | | | 8,010 | | | |
| R sqaured | 0.171 | | | | 0.135 | | | |
| *Source:* FReDA Panel, wave 1 (autumn/winter 2021/22). Authors’ own calculations. | | | | | | | | |

**Table A18 |** Regression tables for Emotional Loneliness and the Interactions with Predictors and Age or Gender

|  | dy/dx | p-value | 95 Confidence Interval | |
| --- | --- | --- | --- | --- |
| **Emotional Loneliness** | | | | |
| *Depressed Mood x Age* | | | | |
| Ref.: No Depressed Mood | | | | |
| 1. Depressed Mood | | | | |
| 18-25 | 0.228 | 0.000 | 0.137 | 0.319 |
| 26-30 | 0.262 | 0.000 | 0.149 | 0.376 |
| 31-35 | 0.160 | 0.011 | 0.037 | 0.282 |
| 36-40 | 0.215 | 0.001 | 0.087 | 0.343 |
| 41-45 | 0.237 | 0.001 | 0.096 | 0.378 |
| 46-51 | 0.184 | 0.017 | 0.033 | 0.334 |
| *Life Satisfaction x Age* | | | | |
| 18-25 | -0.108 | 0.000 | -0.129 | -0.087 |
| 26-30 | -0.125 | 0.000 | -0.148 | -0.102 |
| 31-35 | -0.125 | 0.000 | -0.147 | -0.103 |
| 36-40 | -0.124 | 0.000 | -0.146 | -0.102 |
| 41-45 | -0.107 | 0.000 | -0.130 | -0.084 |
| 46-51 | -0.106 | 0.000 | -0.130 | -0.083 |
| *Partnership Status x Age* |  |  |  |  |
| Ref.: Single |  |  |  |  |
| Living Apart Together |  |  |  |  |
| 18-25 | -0.087 | 0.042 | -0.171 | -0.003 |
| 26-30 | -0.124 | 0.072 | -0.259 | 0.011 |
| 31-35 | -0.156 | 0.054 | -0.315 | 0.003 |
| 36-40 | -0.186 | 0.047 | -0.369 | -0.002 |
| 41-45 | -0.245 | 0.016 | -0.445 | -0.046 |
| 46-51 | -0.263 | 0.009 | -0.462 | -0.065 |
| Cohabitation |  |  |  |  |
| 18-25 | -0.098 | 0.044 | -0.192 | -0.003 |
| 26-30 | -0.204 | 0.000 | -0.302 | -0.106 |
| 31-35 | -0.219 | 0.000 | -0.320 | -0.118 |
| 36-40 | -0.180 | 0.001 | -0.288 | -0.072 |
| 41-45 | -0.142 | 0.014 | -0.256 | -0.029 |
| 46-51 | -0.162 | 0.008 | -0.282 | -0.042 |
| *Social Network x Gender* |  |  |  |  |
| Ref.: No Social Network |  |  |  |  |
| Social Network |  |  |  |  |
| Male | -0.037 | 0.136 | -0.086 | 0.012 |
| Female | -0.091 | 0.000 | -0.138 | -0.045 |

*Source:* FReDA Panel, wave 1 (autumn/winter 2021/22). Authors’ own calculations.

**Table A19 |** Regression tables for Social Loneliness and the Interactions with Predictors Age and Gender

|  | dy/dx | p-value | 95 Confidence Interval | |
| --- | --- | --- | --- | --- |
| **Social Loneliness** | | | | |
| *Partnership Status x Age* | | | | |
| Ref.: Single |  |  |  |  |
| Living Apart Together |  |  |  |  |
| 18-25 | -0.087 | 0.042 | -0.171 | -0.003 |
| 26-30 | -0.124 | 0.072 | -0.259 | 0.011 |
| 31-35 | -0.156 | 0.054 | -0.315 | 0.003 |
| 36-40 | -0.186 | 0.047 | -0.369 | -0.002 |
| 41-45 | -0.245 | 0.016 | -0.445 | -0.046 |
| 46-51 | -0.263 | 0.009 | -0.462 | -0.065 |
| Cohabitation |  |  |  |  |
| 18-25 | -0.098 | 0.044 | -0.192 | -0.003 |
| 26-30 | -0.204 | 0.000 | -0.302 | -0.106 |
| 31-35 | -0.219 | 0.000 | -0.320 | -0.118 |
| 36-40 | -0.180 | 0.001 | -0.288 | -0.072 |
| 41-45 | -0.142 | 0.014 | -0.256 | -0.029 |
| 46-51 | -0.162 | 0.008 | -0.282 | -0.042 |
| *Migration Background x Age* | |  |  |  |
| Ref.: No Migration Background | |  |  |  |
| 1^st^ generation migrant |  |  |  |  |
| 18-25 | 0.292 | 0.006 | 0.084 | 0.501 |
| 26-30 | 0.359 | 0.000 | 0.169 | 0.549 |
| 31-35 | 0.362 | 0.000 | 0.221 | 0.503 |
| 36-40 | 0.314 | 0.000 | 0.170 | 0.459 |
| 41-45 | 0.293 | 0.000 | 0.133 | 0.454 |
| 46-51 | 0.216 | 0.020 | 0.034 | 0.397 |
| 2^nd^ generation migrant |  |  |  |  |
| 18-25 | 0.040 | 0.502 | -0.077 | 0.157 |
| 26-30 | 0.092 | 0.237 | -0.060 | 0.244 |
| 31-35 | 0.245 | 0.002 | 0.089 | 0.401 |
| 36-40 | 0.045 | 0.586 | -0.116 | 0.205 |
| 41-45 | -0.057 | 0.491 | -0.218 | 0.104 |
| 46-51 | 0.103 | 0.212 | -0.059 | 0.264 |
| Social Network x Age |  |  |  |  |
| Ref.: No Social Network |  |  |  |  |
| 18-25 | -0.387 | 0.000 | -0.481 | -0.294 |
| 26-30 | -0.322 | 0.000 | -0.425 | -0.219 |
| 31-35 | -0.333 | 0.000 | -0.425 | -0.242 |
| 36-40 | -0.340 | 0.000 | -0.433 | -0.247 |
| 41-45 | -0.271 | 0.000 | -0.371 | -0.171 |
| 46-51 | -0.274 | 0.000 | -0.377 | -0.170 |
| *Migration Status x Gender* |  |  |  |  |
| Ref.: No Migration Background | | | | |
| 1. Generation Migrant |  |  |  |  |
| Male | 0.265 | 0.000 | 0.160 | 0.370 |
| Female | 0.344 | 0.000 | 0.255 | 0.433 |
| 2. Generation Migrant |  |  |  |  |
| Male | 0.043 | 0.353 | -0.047 | 0.132 |
| Female | 0.102 | 0.015 | 0.020 | 0.183 |

*Source:* FReDA Panel, wave 1 (autumn/winter 2021/22). Authors’ own calculations.

**Table A20 |** Comparison of included and excluded individuals in our sample

|  | Full Sample | | Excluded individuals | | Included individuals | | t-test | | Cohen’s d |
| --- | --- | --- | --- | --- | --- | --- | --- | --- | --- |
|  | (1) | | (2) | | (3) | | (4) | | (5) |
|  | mean | sd | mean | sd | mean | sd | b | t |  |
| Social Loneliness | 1.49 | 1.26 | 1.61 | 1.27 | 1.45 | 1.25 | -0.16^***^ | (-8.40) | -0.13 |
| Emotional Loneliness | 1.49 | 1.09 | 1.64 | 1.10 | 1.43 | 1.08 | -0.20^***^ | (-12.04) | -0.18 |
| Age Categories |  |  |  |  |  |  |  |  |  |
| 18-25 | 0.20 | 0.40 | 0.21 | 0.41 | 0.20 | 0.40 | -0.01 | (-1.81) |  |
| 26-30 | 0.15 | 0.36 | 0.15 | 0.36 | 0.15 | 0.36 | 0.00 | (0.42) |  |
| 31-35 | 0.19 | 0.39 | 0.19 | 0.39 | 0.19 | 0.39 | -0.01 | (-1.07) |  |
| 36-40 | 0.18 | 0.38 | 0.17 | 0.37 | 0.18 | 0.38 | 0.01 | (1.62) |  |
| 41-45 | 0.15 | 0.36 | 0.15 | 0.36 | 0.15 | 0.36 | 0.00 | (0.78) |  |
| 46-51 | 0.14 | 0.34 | 0.14 | 0.34 | 0.14 | 0.34 | 0.00 | (0.28) |  |
| Sex | 1.56 | 0.50 | 1.58 | 0.51 | 1.55 | 0.50 | -0.02^**^ | (-2.84) | -0.04 |
| Satisfaction with HH-Income | 4.51 | 1.23 | 4.32 | 1.28 | 4.59 | 1.20 | 0.27^***^ | (13.87) | 0.22 |
| Educational Background: |  |  |  |  |  |  |  |  |  |
| low | 0.04 | 0.19 | 0.06 | 0.23 | 0.03 | 0.17 | -0.03^***^ | (-9.43) | -0.16 |
| middle | 0.44 | 0.50 | 0.47 | 0.50 | 0.43 | 0.50 | -0.04^***^ | (-4.43) | -0.08 |
| high | 0.52 | 0.50 | 0.48 | 0.50 | 0.54 | 0.50 | 0.06^***^ | (7.94) | 0.12 |
| Migration Background |  |  |  |  |  |  |  |  |  |
| no migration background | 0.78 | 0.42 | 0.72 | 0.45 | 0.80 | 0.40 | 0.08^***^ | (12.07) | 0.19 |
| 1. generation migrant | 0.11 | 0.31 | 0.16 | 0.37 | 0.09 | 0.28 | -0.07^***^ | (-14.69) | -0.23 |
| 2. generation migrant | 0.11 | 0.32 | 0.12 | 0.33 | 0.11 | 0.32 | -0.01 | (-1.50) |  |
| Kids | 0.49 | 0.50 | 0.50 | 0.50 | 0.48 | 0.50 | -0.02^*^ | (-2.12) | -0.04 |
| Partnership Status |  |  |  |  |  |  |  |  |  |
| Single | 0.24 | 0.43 | 0.23 | 0.42 | 0.24 | 0.43 | 0.01 | (1.88) |  |
| LAT | 0.13 | 0.33 | 0.14 | 0.35 | 0.12 | 0.33 | -0.02^***^ | (-3.64) | -0.06 |
| Cohabitation | 0.63 | 0.48 | 0.63 | 0.48 | 0.64 | 0.48 | 0.01 | (0.86) |  |
| Social Network | 0.62 | 0.48 | 0.61 | 0.49 | 0.63 | 0.48 | 0.02^**^ | (2.64) | 0.04 |
| Anxiety Disorders | 0.11 | 0.31 | 0.11 | 0.31 | 0.11 | 0.31 | -0.00 | (-0.20) |  |
| Depressed Mood | 1.76 | 0.73 | 1.78 | 0.74 | 1.76 | 0.73 | -0.02 | (-1.64) |  |
| Life Satisfaction | 7.24 | 1.84 | 7.17 | 1.89 | 7.26 | 1.82 | 0.09^**^ | (2.98) | 0.05 |
| Covid19: Positive Aspects | 3.53 | 1.12 | 3.46 | 1.14 | 3.55 | 1.11 | 0.10^***^ | (5.51) | 0.09 |
| Covid19: Personal Burden | 3.23 | 1.16 | 3.33 | 1.15 | 3.20 | 1.16 | -0.13^***^ | (-7.12) | -0.11 |
| Typ 2 Diabetes | 0.03 | 0.16 | 0.03 | 0.16 | 0.02 | 0.16 | -0.00 | (-0.86) |  |
| Hypertonie | 0.10 | 0.30 | 0.09 | 0.28 | 0.10 | 0.30 | 0.01^*^ | (2.40) | 0.03 |
| Cancer | 0.02 | 0.14 | 0.02 | 0.14 | 0.02 | 0.14 | -0.00 | (-0.22) |  |
| Asthma | 0.09 | 0.29 | 0.09 | 0.28 | 0.09 | 0.29 | 0.00 | (1.07) |  |
| Obesity | 0.17 | 0.37 | 0.19 | 0.39 | 0.16 | 0.37 | -0.03^***^ | (-4.41) | -0.08 |
| Neuroticism | 2.95 | 0.64 | 2.97 | 0.64 | 2.95 | 0.64 | -0.02 | (-1.72) |  |
| Openness to Experience | 3.34 | 0.79 | 3.34 | 0.78 | 3.35 | 0.79 | 0.01 | (0.52) |  |
| Agreeableness | 3.66 | 0.72 | 3.68 | 0.73 | 3.66 | 0.72 | -0.03 | (-1.69) |  |
| Conscientiousness | 3.65 | 0.78 | 3.60 | 0.78 | 3.66 | 0.77 | 0.05^**^ | (3.04) | -0.03 |
| Extraversion | 3.06 | 0.75 | 3.08 | 0.74 | 3.06 | 0.75 | -0.03 | (-1.60) |  |
| Observations | 20206 |  | 5763 |  | 14443 |  | 20206 |  |  |

Source: FReDA Panel, wave 1 (autumn/winter 2021/22). Authors’ own calculations.

**Table A21:** Comparison of the results obtained from the OLS and ordered logit models.

|  | (1) | (2) | (3) | (4) |
| --- | --- | --- | --- | --- |
|  | OLS  Social Loneliness | Ordered Logit   Social Loneliness | OLS Emotional Loneliness | Ordered Logit  Emotional Loneliness |
| Age 18-25 | 1.277^***^ | 1.256^***^ | 1.509^***^ | 1.495^***^ |
|  | (48.19) | (44.53) | (67.74) | (63.54) |
|  |  |  |  |  |
| Age 26-30 | 1.312^***^ | 1.298^***^ | 1.477^***^ | 1.465^***^ |
|  | (50.07) | (46.13) | (67.07) | (63.73) |
|  |  |  |  |  |
| Age 31-35 | 1.454^***^ | 1.448^***^ | 1.453^***^ | 1.433^***^ |
|  | (64.08) | (58.33) | (76.21) | (71.53) |
|  |  |  |  |  |
| Age 36-40 | 1.522^***^ | 1.516^***^ | 1.387^***^ | 1.366^***^ |
|  | (64.00) | (58.82) | (69.42) | (65.06) |
|  |  |  |  |  |
| Age 41-45 | 1.570^***^ | 1.574^***^ | 1.367^***^ | 1.335^***^ |
|  | (60.29) | (55.20) | (62.44) | (57.37) |
|  |  |  |  |  |
| Age 46-51 | 1.597^***^ | 1.600^***^ | 1.369^***^ | 1.335^***^ |
|  | (57.18) | (52.13) | (58.33) | (53.50) |
| Observations | 14443 | 14443 | 14443 | 14443 |

*t* statistics in parentheses

^*^ *p* < 0.10, ^**^ *p* < 0.05, ^***^ *p* < 0.01

Source: FReDA Panel, wave 1 (autumn/winter 2021/22). Authors’ own calculations.

**Figure A1** **|** Base Model for Overall Loneliness, Emotional Loneliness and Social Loneliness on a y-axis ranging from 0 – 3

*Note:* The base model includes age (categorical) and gender (binary). Loneliness was measured on a scale from 0 to 3. Figure 1 in the main text presents a zoomed-in view of the graph, with the y-axis ranging from 1.2 to 1.8.

*Source:* FReDA Panel, wave 1 (autumn/winter 2021/22). Authors’ own calculations.

**Figure A2 |** Full Model for Overall Loneliness, Emotional Loneliness and Social Loneliness on a y-axis ranging from 0 – 3

*Note:* Full model including age (categorical) and gender (binary), and all predictor sets from Models 1-4: socio-demographic characteristics, social relationships, health variables, and personality traits. Loneliness was measured on a scale from 0 to 3. Figure 3 in the main text presents a zoomed-in view of the graph, with the y-axis ranging from 1.2 to 1.8.

*Source:* FReDA Panel, wave 1 (autumn/winter 2021/22). Authors’ own calculations.

**Figure A3 |** Full Model for Overall Loneliness, Emotional Loneliness and Social Loneliness on a y-axis ranging from 0 – 3, stratified by gender

*Note:* Full model including age (categorical) and gender (binary), and all predictor sets from Models 1-4: socio-demographic characteristics, social relationships, health variables, and personality traits. Loneliness was measured on a scale from 0 to 3. Figure 4 in the main text presents a zoomed-in view of the graph, with the y-axis ranging from 1.2 to 1.8.

*Source:* FReDA Panel, wave 1 (autumn/winter 2021/22). Authors’ own calculations.
